# Adaptation of a clinical practice guideline for home care nursing in heart failure patients: a multi-method study

**DOI:** 10.1186/s12912-026-04774-x

**Published:** 2026-07-04

**Authors:** Leila Hashemlu, Roghayeh Esmaeili, Fatemeh Bahramnezhad, Camelia Rohani

**Affiliations:** 1 Non-Communicable Diseases Research Center, Qazvin University of Medical Sciences, Qazvin, Iran; 2 Department of Medical-Surgical Nursing, School of Nursing and Midwifery, Shahid Beheshti University of Medical Sciences, Vali-Asr Avenue, Cross of Vali-Asr and Neiaiesh Highway, Opposite to Rajaee Heart Hospital, Tehran, 1996835119 Iran; 3 Department of ICU Nursing, School of Nursing & Midwifery, Nursing and Midwifery Care Research Center, Tehran University of Medical Sciences, Tehran, Iran; 4 Independent Researcher, Stockholm, Sweden; 5 Department of Community Health Nursing, School of Nursing & Midwifery, Shahid Beheshti University of Medical Sciences, Vali-Asr Avenue, Cross of Vali-Asr and Neiaiesh Highway, Opposite to Rajaee Heart Hospital, Tehran, 1996835119 Iran

**Keywords:** Clinical practice guideline, Adaptation, Home care, Heart failure, Nursing

## Abstract

**Background:**

The demand for home healthcare services for chronic conditions has increased due to the transition of care from hospitals to the home. This study aimed to adapt a Clinical Practice Guideline (CPG) for home-based nursing care of adult patients with heart failure in the healthcare system of Iran.

**Methods:**

This study was conducted using a multi-method design within the 10-step framework of the Practice Guidelines Evaluation and Adaptation Cycle (PGEAC). A systematic review was conducted in PubMed, Scopus, Web of Science, and nine publicly accessible CPG databases to find relevant guidelines. Subsequently, a robust CPG was selected based on the Appraisal of Guidelines for Research and Evaluation-II (AGREE-II) checklist. After translating the guideline, a search for supporting evidence was conducted in databases for each guideline recommendation, and the results were classified based on the level of evidence. Next, a qualitative study was conducted to strengthen recommendations with a weak evidence level and uncover additional recommendations and domains. The preliminary draft of the CPG was then evaluated in an expert panel meeting. In the final phase, the adapted CPG was validated through three Delphi rounds.

**Results:**

“Adapting Heart Failure Guidelines for Nursing Care in Home Health Settings,” was selected as an appropriate CPG with 145 recommendations within five domains. Following translation and evaluation of the evidence level, 26 recommendations were categorized in the weak level of evidence. In the qualitative phase, these recommendations were further examined, and also 26 new recommendations were developed; 12 under the domain of the “Generic care in heart failure” and 14 within two newly identified domains. Twenty-one recommendations were removed during the expert panel meeting, as well as 33 ones in the first Delphi round, due to duplication or not being applicable the Iranian healthcare system. The final adapted CPG included 117 recommendations within six domains.

**Conclusions:**

This adapted CPG provides a robust framework for nurses and other healthcare professionals to deliver home healthcare services for Iranian heart failure patients and their families. It can enhance the quality of home-based care, reduce hospital readmissions, improve patients’ outcomes, and the cost-effectiveness of home-based care programs.

**Supplementary Information:**

The online version contains supplementary material available at https://doi.org/10.1186/s12912-026-04774-x.

## Introduction

Cardiovascular diseases are among the most prevalent chronic conditions and represent the leading cause of mortality and disability in most countries worldwide [1]. Among cardiovascular diseases, heart failure is the most common and persistent disorder and plays an important role in healthcare systems [2]. As the disease progresses, symptoms of heart failure, including dyspnea, pain, anxiety, fatigue, and depression, may become more severe and resistant to treatments. Therefore, patients experience poor Quality of Life, and require acute care, and endure suffering during the end of life [3].

In Iran, the incidence of heart failure among hospitalized elderly patients has been reported at 7.7% to 8.5%, which is higher than in other countries of the region. Heart failure in Iran is considered one of the primary causes of disability and death. With demographic transitions and an aging population, its current prevalence - estimated at 3500 cases per 100,000 individuals - is expected to rise in the near future [4]. Targeted interventions can reduce the risk of readmission, healthcare costs, financial burdens on families, and slowing disease progression to lessen the economic, social, and psychological burden of these outcomes.

Patients with heart failure experience various adverse outcomes, including hospital readmission, poor self-care, reduced Quality of Life, and low self-efficacy [5]. The economic, social, and psychological burden of these outcomes can be decreased through targeted interventions to reduce the risk of readmission, healthcare costs, financial burdens on families, and slowing disease progression [6]. Numerous studies have demonstrated that management-based nursing care significantly reduces hospital readmission rates in patients with heart failure [7–9].

Evidence shows that heart failure is a global health priority and patients should receive team-based care to improve treatment adherence, reduce hospital readmissions, and increase survival. Among different models of care worldwide for patients with heart failure, home healthcare models constitute a fundamental component of interdisciplinary team-based care. Innovation in home healthcare models with early intervention can improve patient outcomes such as lifestyle modification and increasing Quality of Life [6].

Despite the growing importance of home healthcare services for patients with chronic diseases, no uniform standard process or instruction for delivering home healthcare services is implemented in the healthcare system of Iran [10], and the structure of these services is still evolving in the country [11]. Additionally, home healthcare services are not integrated to the Primary Health Care (PHC) network of the country as a national network [12]. Statistics show that only 2% of patients with heart failure utilize home healthcare services. However, hospitalizations still account for 68% of the total medical expenses [13]. Furthermore, family caregivers are predominantly involved in caring for patients at home, while general caring services and specialized healthcare services are provided in a heterogeneous manner and outside of insurance coverage [14]. The majority of home healthcare services are currently provided by the private sector and sometimes by special units in hospitals affiliated with medical universities [10]. This situation may affect the quality of care and highlights the need to consider the contextual realities of Iran when designing and implementing home healthcare interventions.

Although a variety of healthcare professionals provide healthcare services in home settings, nurses are the cornerstone of this care program and play a key role in managing and coordinating home healthcare services [15]. Given nurses’ comprehensive understanding of patients’ problems and challenges, they can work closely with patients and their families. They play a pivotal role in developing care, treatment, education, and rehabilitation programs [16].

Despite the increasing focus on home healthcare services and ongoing initiatives to reduce healthcare costs while improving patient care quality, recent international reports indicate that home healthcare nurses often lack sufficient knowledge to manage patients with heart failure at home [17]. Moreover, although Clinical Practice Guidelines (CPGs) for patients with heart failure are widely available, their application in home healthcare nursing remains complex. Many of these guidelines are developed primarily for physicians, who hold prescribing authority, and therefore cannot be directly applied by nurses in home healthcare settings. For example, guidelines published by the American College of Cardiology (ACC), or the American Heart Association (AHA) have not been adequately integrated into home healthcare nursing programs for patients with heart failure [18]. Therefore, access to specific and appropriate home healthcare CPGs is essential, and such guidelines must be clear, explicit, and specifically relevant to nursing practice [19]. Evidence shows that some recommendations in international guidelines may not be directly applicable to other settings due to their dependence on advanced healthcare systems, well-defined support roles, and differences in cultural and demographic characteristics. Therefore, applying these guidelines without contextual adaptation may lead to misalignment with practical care processes and create different challenges for healthcare providers [20].

Around the world, various programs have been expanded to develop and adapt CPGs in accordance with national and state-level approaches in each country. Depending on the context and available evidence, it is recommended to develop a new clinical guideline or adapt an existing guideline. When sufficient evidence on the subject of interest is lacking, or when the subject is closely linked to the cultural context of a society, the development of a new gudeline is preferred [21]. But, the concept of guideline adaptation originates from the recognition that, in most fields, various strategies have already been developed globally. It is therefore sufficient to select the most credible strategy and adapt it to the target region [22]. Guideline adaptation is the result of a shift from applying the same evidence-based recommendations everywhere to applying the same evidence, but in harmony with the cultural and regional requirements of the target population. By adapting CPGs to local settings, it is possible to avoid rework and maximize resource utilization [23]. Thus, by focusing on the limitations existing in the healthcare system of Iran and society in the areas of stewardship, financing, human resources and equipment, as well as providing home healthcare services [10], the adaptation process is justified in this study.

In the present study the Practice Guidelines Evaluation and Adaptation Cycle (PGEAC) was used as a framework for CPG adaptation. The PGEAC was created by two Canadian researchers, Graham and Harrison, in 2005 [24]. It offers a framework to organize and decide how to adapt high-quality guidelines. The PGEAC framework is designed to make it easier to compare different guidelines and recommendations on the same subject and provide a systematic method for evaluating the quality of guidelines and clinical tools. It emphasizes that recommendations should not only be based on evidence but also aligned with each country’s available resources, professional roles, and implementation capacity [23].

Accordingly, the aim of this study was to adapt a valid international clinical guideline for home healthcare of patients with heart failure in order to develop a version that is compatible with Iran’s cultural context, healthcare delivery system, and healthcare system capacities. In a comprehensive search, the authors found no existing CPG for home healthcare of patients with heart failure that could be applied by Iranian nurses. Thus, the present study was conducted with the aim of adapting a strong CPG for home-based nursing care of adult patients (18 years old and over) with heart failure within the healthcare system of Iran.

## Methods

**Fig. 1:**
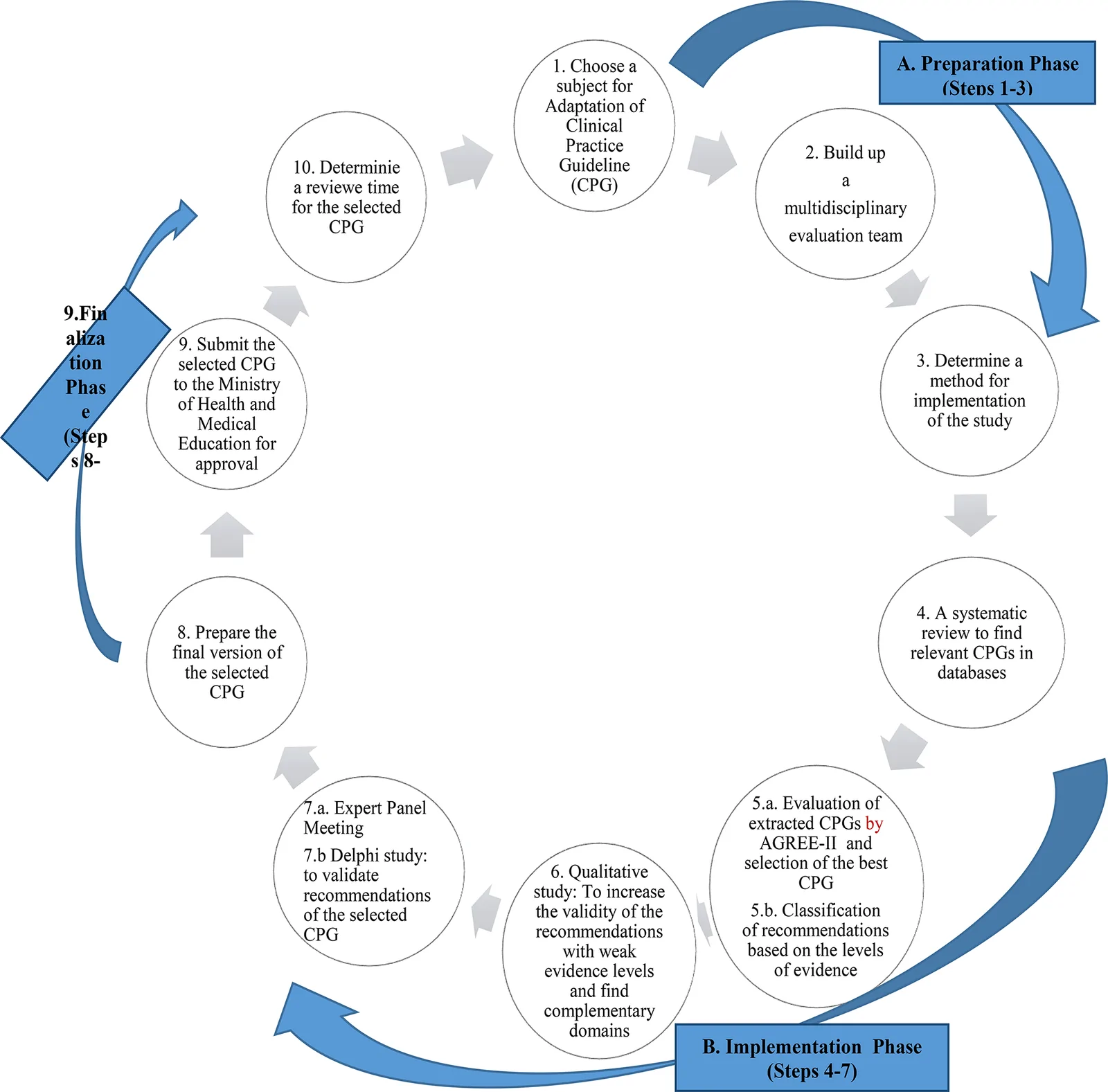
The 10-step practice guidelines evaluation and adaptation cycle (PGEAC) for guideline adaptation

The present study followed a multi-method design, including a systematic review of CPGs, a qualitative study, an expert panel meeting and a Delphi study. It was conducted from May 2019 to February 2022 within the framework of the PGEAC [24]. The PGEAC constitutes a 10-step cycle for adaptation of a guideline, and divides into three main phases: Preparation phase (Steps 1–3), Implementation phase (Steps 4–7), and Finalization phase (Steps 8–10) (Fig. 1).

### Preparation phase of the PGEAC (Steps 1–3)

This phase involves identifying the needs of the target groups, choosing a subject for adaptation of the CPG, building up a multidiciplinary evaluation team, and determining a method for implemention of the study. During the preparation phase, the research team consulted to assess the necessity of adapting an appropriate CPG for home-based care of adult patients with heart failure within the healthcare system of Iran. Next, based on the significance of the subject and its impact on clinical outcomes and efficiency, relevant resources were prioritized. A multidisciplinary team was formed to guide and implement the guideline adaptation process. The scope of the study was then defined, including formulating clinical questions, determining the target population and objectives of guideline adaptation, as well as selecting a tool to evaluate existing guidelines.

### Implementation phase of the PGEAC (Steps 4–7)

The Implementation phase included conducting a systematic review to find relevant CPGs in different databases, evaluation and selection of a specific guideline, subsequently searching the databases to find evidence for each of the selected guideline recommendations and classification of them based on the level of evidence. Then, followed by conducting a qualitative study, an expert panel meeting, and a Delphi study. Multiple data sources were utilized during this phase, as detailed below.

#### Step 4. the systematic review of CPGs

In this step for selection of an appropriate CPG based on inclusion and exclusion criteria, a systematic review was conducted in PubMed, Web of Science, Scopus, and nine public available CPG databases, including “*Agency for Healthcare Research and Quality & National Guideline Clearinghouse*,” “*Guideline International Network (G-I-N)*,” “*New Zealand Guidelines Group*,” “*National Health and Medical Research Council (NHMRC)*,” “*National Institute for Clinical Excellence (NICE; UK)*,” “*Australian National Health and Medical Research Council*,” “*Scottish Intercollegiate Guidelines Network (SIGN)*,” “*Guidelines and Clinical Practice Update Library (Canadian Cardiovascular Society: CCS)*,” “*International Council of Cardivascular Prevention and Rehabilitation (ICCPR)*.” The inclusion criteria were: the guideline was specifically developed for patients with heart failure, and its publication language was English. It was published between 1st of January 2000 and 17th of May 2021, with the label of guideline, recommendation, guideline adherence or practice guideline. When there were multiple versions of the guideline, the most recently updated one was chosen. The exclusion criteria were: the guideline did not refer to home healthcare services, and was not supported by a public or private healthcare association, organization, society, or government agency. Also, its recommendations were not based on a systematic review of the literature.

Two members of the research team independently screened the titles and abstracts of the selected records to identify those CPGs relevant to the study. This systematic review was conducted and reported elsewhere [19] in accordance with the 2020 Preferred Reporting Items for Systematic reviews and Meta-Analyses (PRISMA) statement.

#### Step 5. a: evaluation of extracted CPGs using the AGREE-II

This step involved the evaluation of the extracted CPGs using the Appraisal of Guidelines for Research & Evaluation (AGREE-II) checklist [25] by members of the research team in terms of novelty, publication date, cited sources, frequency of updates, and overall content. Based on this evaluation, the best CPG with the highest score was selected for adaptation. The selected CPG was then translated from English into Persian using the Brislin method [26]. After that a “Guideline Matrix” was prepared to examine the level of evidence supporting each recommendation of the guideline.

#### Step 5. b: classification of recommendations based on the level of evidence

In this step, for each recommendation of the selected CPG, relevant and credible evidence was systematically searched in PubMed, Scopus, and Web of Science. Subsequently, in accordance with the Heart Failure Society of America (HFSA) guideline, the CPG recommendations were classified into levels of A, B, and C, representing strong, moderate, and weak evidence, respectively (Supplementary File 1).

#### Step 6: the qualitative study

A descriptive qualitative study was conducted using directed content analysis method to validate recommendations with low levels of evidence in the selected CPG, and also to identify complementary domains with new recommendations. The qualitative data analysis was implemented using Shannon and Hsieh (2005) approach [27]. This approach was selected due to its ability to link new data to the conceptual framework of the selected guideline.

Data were collected through individual, semi-structured, and in-depth interviews with 25 stakeholders using an interview guide (Supplementary File 2). Data collection continued until data saturation, and then three additional interviews were conducted to ensure reproducibility and comprehensiveness of the data. To establish the trustworthiness of qualitative data, the four criteria proposed by Lincoln and Guba; credibility, dependability, confirmability, and transferability were applied [28]. Credibility was ensured through prolonged engagement with the data, establishing appropriate record from participants, dedicating sufficient time to data collection and analysis, conducting reviews of interviews and extracting codes and categories in the research team. Additionally, to assess the consistency of findings and enhance dependability, the interviews and extracted codes were returned to three participants for verification of their perspectives. To achieve confirmability, all stages of the qualitative research, particularly data analysis procedures, were thoroughly documented. Moreover, the analyses conducted on the transcripts of interviews, including extracted codes, categories and subcategories were independently evaluated by two external referees (both faculty members from two nursing schools holding doctoral degrees in nursing and experts in qualitative research). Transferability was addressed by ensuring diversity in participants and providing detailed descriptions of them, thereby facilitating the applicability of findings to similar context. MAXQDA software version 10 was used to manage and analyze the qualitative data.

#### Step 7. a: the expert panel meeting

At first, the initial draft of the adapted CPG was prepared by the research team, incorporating recommendations extracted from the selected guideline as well as those derived from the qualitative phase. Subsequently, an expert panel comprising six specialists in heart failure and home care participated in a two-hour online meeting via Skype under the leadership of the first author (L.H). The panel was tasked with evaluating the recommendations and assessing their alignment with the cultural and social context of patients and families in our national healthcare system.

The panel focused on recommendations with low levels of evidence and those derived from the qualitative phase. A questionnaire containing demographic information questions, an informed consent form, a set of recommendations, and an evidence matrix was sent to the experts 10 days before the meeting to allow them sufficient time to review the guideline. The revised version of the CPG, was then finalized for entry into the Delphi phase.

#### Step 7. b: the delphi study

In this phase, the classical Delphi with three rounds was run. It was aimed to achieve a consensus among the experts, and validate the recommendations. The overall structure of each round was as follows:

##### Round 1

A table containing all revised recommendations during the expert panel meeting was sent by email to a group of specialists (*n* = 33) to provide their opinions on the CPG recommendations. Specialists were asked to provide qualitative feedback by freely commenting on content and wording of each recommendation.

*Round 2*: Following the first round, a revised version of the CPG was sent to the same specialists. They were asked to provide additional qualitative comments for each recommendation and to assess the Content Validity Ratio (CVR) of the recommendations with low levels of evidence (C-Level). Each recommendation was rated on a three-point Likert Scale: “not necessary,” “useful but not essential,” and “essential,” with scores ranging from 0 to 2. The minimum acceptable CVR value was estimated according to Lawshe’s Table [29]. “The formula for calculating the CVR is CVR = (Ne - N/2) / (N/2), where Ne is the number of specialists who rated an item as “essential,” and N is the total number of specialists participating in the evaluation” [30].

##### Round 3

In the third round, the adapted CPG recommendations resulting from round two, together with the AGREE-II, were sent through email to the specialists. Detailed instructions regarding the AGREE-II and the scoring process were provided alongside the questionnaire.

### Finalization phase of the PGEAC (Steps 8–10)

In this phase, the final draft of the adapted CPG for home care of patients with heart failure was produced and submitted to the Ministry of Health and Medical Education to approve within the healthcare system of the country. A three-year period was established for the guideline’s update and revision.

## Results

The findings of the study, are organized into three phases of the PGEAC: Preparation (Steps 1–3), Implementation (steps 4–7), and Finalization (Steps 8–10).

### Results of the preparation phase (Steps 1–3)

In this phase, the CPG evaluation team, consisting of three faculty members and one doctoral student in nursing from two medical universities was made. They assessed and prepared a report for the necessity of adapting a valid CPG for home care in adult patients with heart failure in the healthcare system of Iran. Also, existing resources were reviewed and prioritized. Furthermore, the AGREE-II checklist was selected to evaluate the available CPGs.

### Results of the implementation phase (Steps 4–7)

The findings of this phase included the results of the systematic review, selection of the best CPG and translation, classification of the levels of evidence for the recommendations of the selected CPG, qualitative study results, the results of the expert panel meeting, and the Delphi study.

#### Results of the systematic review of CPGs

According to the inclusion criteria, 22 relevant CPGs were identified through a systematic review of existing records in different platforms and databases. After screening and detailed evaluation of the records by two authors independently based on the inclusion criteria, 10 CPGs were selected. They included two specific home care guidelines and eight general guidelines. Next, using the AGREE-II, the CPG entitled “Adapting Heart Failure Guidelines for Nursing Care in Home Health Settings” was selected for adaptation. This CPG represents an adapted version of the general heart failure guideline, specified for home care setting [31].

The selected CPG comprised 145 recommendations across five domains, including “Generic care in heart failure,” “Heart failure in minority population,” “Heart failure with preserved ejection fraction,” “Heart failure with reduced ejection fraction,” and “Heart failure with comorbidities.” It was selected among 10 CPGs based on scoring 0 to 100 on the AGREE-II. This guideline scores achieved above 60 in four key domains of the AGREE-II (Domain 1: Scope and purpose, Domain 2: Stakeholder involvement, Domain 3: Rigor of development, Domain 4: Clarity of presentation), and above 50 in the remaining domains (Domain 5: Applicability and Domain 6: Editorial independence). The results of this step have been previously published [19].

The selected CPG was translated from English into Persian using the method of Brislin [26]. Following translation, a guideline matrix was prepared to assess the level of evidence for the 145 recommendations. For each recommendation, relevant evidence was systematically searched in PubMed, Scopus, and Web of Science, using the PICO (Patient/Population, Intervention, Comparison, Outcomes) framework. The level of evidence for recommendations showed that there are A (strong, *n* = 49), B (moderate, *n* = 70), and C (weak, *n* = 26) levels after classification by the HFSA guideline.

#### Results of the qualitative study

A descriptive qualitative study with the aim of exploring “Stakeholders’ experiences and perceptions of the needs of patients with heart failure and their families for delivering healthcare services at home” was conducted. Participants were 25 stakeholders, including nurses (*n* = 7), cardiologists and general practitioners (*n* = 4), nutritionists (*n* = 2), psychologists (*n* = 3), family caregivers (*n* = 6), and adult patients with heart failure (*n* = 3). This study was carried out to validate the selected CPG recommendations with a low level of evidence, and to develop new recommendations.

In this study, directed content analysis was used based on the five domains of the selected CPG. However, the interview questions were purposefully focused on recommendations with a low level of evidence. The results showed that most of the extracted codes were classified under the domain of “Generic care in heart failure,” and the rest of domains were not reflected independently. From 1520 initial codes, similar ones were merged, resulting in 1067 final codes, which were subsequently categorized into 52 subcategories, nine categories, and three overarching themes aligned with the classification of the domains of selected CPG (Table 1). Within the categories of the main theme; “Generic care in heart failure,” a series of codes were reflected (12 recommendatios were extracted). Additionally, two new themes; “Family caregivers’ needs” and “Standardized home care system” emerged (14 recommendations were extracted). These two new themes, along with newly developed recommendations, were integrated into the selected CPG.

**Table 1:** Results of the qualitative study to explore stakeholders’ experiences and perceptions of the needs of heart failure patients and their families for delivering healthcare services at home

Main Themes	Categories	Subcategories	Quotations
Generic Care in Heart Failure	Patient Assessment	- Skill in history taking and physical examination - Knowledge of diagnostic tests and investigations - Assessment of treatment adherence at home - Documentation of prescribed and self-medicated drugs - Identification and clear documentation of patient needs - Care planning with specific goals for each patient	“During the first assessment session, all medications used by the patient must be recorded to ensure safe care.” (Participant 4, Female Nurse with Doctoral degree, 33 years old)
	Non-pharmacological and Physical Care	- Daily home assessment of patients - Symptom management at home - Management of daily activities - Maintaining a healthy diet - Physical activity based on functional status - Continuous patient education - Linking patients to peer and support groups - Importance of effective communication with family - Sleep hygiene and referral to specialists if necessary - Smoking cessation and healthy lifestyle programs - Ensuring patient safety during home care - Vaccination according to guidelines	“The patient’s weight should be measured daily.” (Participant 15, Male Assistant Professor of Nursing with Master degree, 60 years old)
	Psychological Factors	- Assessment of mental health and changes in social factors - Depression - Feelings of regret and shame - Unclear future perception - Hope and hopelessness - Death anxiety - Acceptance and commitment therapy for death anxiety - Early counseling and referral to psychologists or psychiatrists if needed	“Eventually, thoughts about death come to mind-what will happen to me and my children in the future?” (Participant 22, Male Patient with Bachelor degree, Retired, 64 years old)
	Medication-related Knowledge for Home Care	- Commonly used drugs and contraindications - Polypharmacy - Attention to side effects and their management - Medication errors and error reporting	“When providing care at home, attention to medication safety is vital, especially for cardiac patients who often use multiple medications.” (Participant 3, Female Nurse, Doctoral Student, 35 years old)
	Patient Self-care	- Teaching self-care and decision-making skills - Empowering patients for disease management - Developing self-care plans for comorbid conditions	“Heart failure patients usually have other diseases such as diabetes or arthritis. When providing home care, the nurse must plan for these conditions as well.” (Participant 1, Female Nurse with Bachelor degree, 45 years old)
Family Caregivers’ Needs	Unmet Needs of Family Caregivers	- Emotional needs - Physical needs - Support for timely access to formal and informal services - Need for self-care training for caregivers - Need for safety during home care	“Unfortunately, there is no informational support available. We do not have any training programs for family caregivers. Both patients and their families need guidance to reduce stress and accept the illness.” (Participant 14, Male General Physician, 54 years old)
	Enhancing Family Caregiver Competence	- Training within home care programs - Empowerment of families and patients for effective home care - Emotional support for patients - Family participation in planning and decision-making	“We cannot decide about the patient alone. If care quality matters, the patient and family should be involved in all stages of home care—from planning to implementation and evaluation.” (Participant 2, Female Nurse with Master degree, 44 years old)
Standardized Home Care System	Need for Specialized Home Care Services	- Development of clinical practice guidelines for home care of heart failure patients - Designing educational software and videos - Establishment of a referral system - Use of qualified interdisciplinary professionals - Strong collaboration within the home care team - Continuous and follow-up care - Coordination of home care services - Promoting the culture of home care - Provision of palliative care at home	“If I knew that healthcare providers and doctors were competent, I would have no stress about my patient.” (Participant 16, Female Family Caregiver, illiterate, 52 years old)
	Addressing Structural Barriers to Home Care	- Monitoring and evaluation of home care - Insurance coverage for home care programs - Standardizing the home environment for patient care	“Families face many problems, mainly financial. The cost of home care services is high, and many patients cannot afford them. I wish home care were covered by insurance.” (Participant 1, Female Nurse with Bachelor degree, 45 years old)

#### Results of the expert panel meeting

The specialists in the panel included one cardiologist, three nursing faculty members, one experienced home healthcare nurse, and one researcher specializing in heart failure and home care. Following the integration of new themes and recommendations derived from the qualitative study into the selected CPG, the research team prepared a draft of the adapted CPG, containing 171 recommendations (145 initial recommendations and 26 new recommendations from the qualitative phase) across seven domains: “Generic care in heart failure,” “Heart failure in minority populations,” “Heart failure with preserved ejection fraction,” “Heart failure with reduced ejection fraction,” “Heart failure with comorbidities,” (five old domains),” “Family caregivers’ needs” and “Standardized home care system” (two new domains). This draft was reviewed and evaluated by six expert panel members in a two-hour online meeting via Skype under the leadership of the first author (L.H).

During this meeting, overlapping recommendations were consolidated, and the recommendations were categorized into three main areas: “What nurses should do,” “What nurses should know,” and “What nurses good to know.” Moreover, after discussion and agreement, the target audience of the adapted CPG was expanded beyond nurses, and other home healthcare providers, such as general practitioners, cardiologists, psychologists, and nutritionists, were added. Next, 26 recommendations with low levels of evidence (C-Level), including 11 from the domain of the “Generic care in heart failure,” three from “Heart failure with preserved ejection fraction,” two from “Heart failure in minority populations,” nine from “Heart failure with reduced ejection fraction,” and one from “Heart failure with comorbidities” together with the 26 new recommendations obtained from the qualitative study, were discussed during the expert panel meeting to decide whether to retain or remove them using binary voting (Yes/No). In the end, 21 recommendations were removed because of overlap and lack of applicability to the healthcare system of Iran. These included recommendations numbers 4 and 32 from domain one (Generic care in heart failure), recommendations numbers 1 to 12 from domain two (Heart failure in minority populations), recommendation number 12 from domain three (Heart failure with preserved ejection fraction), recommendations numbers 32, 34, 39, 40, and 53 from domain four (Heart failure with reduced ejectionfraction), and recommendation number 7 from domain five (Heart failure with comorbidities). As a result, domain two (Heart failure in minority populations) was completely removed due to their focus on the patient’s race (targeting African-American patients), overlapping with other domains and lack of applicability to the healthcare system of Iran. Thus, the total number of recommendations decreased from 171 to 150 in the expert panel meeting. This revised draft with 150 recommendations and six domains along with the evidence matrix was then prepared for the first round of the Delphi to validate the adapted CPG recommendations.

#### Results of the delphi study

In this phase, the adapted CPG for home care of adult patients with heart failure was evaluated and validated through a three-round Delphi by specialists. The participants were healthcare providers, researchers, and university faculty members in the field of heart failure and home care.

##### Round 1

Thirty-three specialists were invited to evaluate the recommendations of the adapted CPG, but only 19 specialists responded. The demographic characteristics of them are presented in Table 2. Qualitative feedback was collected and resulted in the removal of 33 recommendations. After the first round, the total number of recommendations decreased from 150 to 117 within six domains.

**Table 2:** Demographic characteristics of specialists in the first to third rounds of the Delphi study

Number	Gender	Age (years)	Education level	Work experience with heart failure patients (years)	Position
1	Female	45	PhD in Nursing	23	PhD in Nursing
2	Female	44	PhD in Nursing	23	PhD in Nursing
3	Female	35	MSc in Nursing	10	Nurse
4	Female	33	PhD in Nursing	8	Faculty Member
5	Female	35	MSc in Nursing	2	Nurse
6	Female	50	PhD in Nursing	14	Faculty Member
7	Female	38	MSc in Nursing	10	Nurse
8	Male	41	MSc in Nursing	13	Nurse
9	Male	44	PhD in Nursing	21	Faculty Member
10	Female	46	Fellowship in Heart Failure*	11	Physician
11	Female	38	Cardiologist*	9	Physician
12	Female	44	Cardiologist*	13	Physician
13	Male	35	Cardiologist	7	Physician
14	Male	54	General Practitioner	27	Physician
15	Male	60	MSc in Nursing	30	Faculty Member
16	Male	49	PhD in Nursing*	28	Faculty Member
17	Male	54	PhD in Islamic Jurisprudence and Foundations of Health-related Quranic and Hadith Studies**	27	Faculty Member
18	Male	38	PhD in Clinical Pharmacy**	6	Pharmacist
19	Female	36	PhD in Clinical Pharmacy**	8	Faculty Member

* Specialists who did not participate in the second and third rounds of the Delphi

** Specialists who did not participate in the third round of the Delphi

##### Round 2

Following the first round, a revised version of the CPG with 117 recommendations in 6 domains was resubmitted to the 19 specialists for further evaluation. Participants were asked to provide qualitative feedback for all recommendations and to assess low evidence-level ones (16 recommendations from the original CPG and 26 new recommendations derived from the qualitative study) using the CVR. After two reminders, only 15 specialists delivered their feedback over a 30-day period. No recommendations were removed in this round, as all evaluated items achieved a CVR greater than 0.59. The minimum acceptable CVR for 15 specialists, based on Lawshe’s Table, was estimated at 0.49 [30]. This CPG version was accepted by all the specialists without any changes being made.

##### Round 3

The revised CPG was then provided to the same 15 specialists, similar to round 2, for evaluation of the CPG using the AGREE-II. During this step, responses were received from only 12 specialists. The results of this round are presented in Table 3.

**Table 3:** Results of the validation of the adapted Clinical Practice Guideline (CPG) for home care of heart failure patients in the third round of the Delphi based on the opinions of 15 specialists by the AGREE-II checklist

Domain 1: Scope and Purpose	Domain 2: Stakeholder Involvement	Domain 3: Rigor of Development	Domain 4: Clarity of Presentation	Domain 5: Applicability	Domain 6: Editorial Independence
100	92	90	80	60	89

The score range for each domain is between 0 and 100

Table 3 shows the validation of the adapted CPG with 117 recommendations using the AGREE-II across six domains of the checklist: “Scope and purpose,” “Stakeholder involvement,” “Rigor of development,” “Clarity of presentation,” “Applicability,” and “Editorial independence.” Based on the domain scores (0-100), the lowest and highest scores were 60 for “Applicability” and 100 for “Scope and purpose,” respectively. But, the scores of other domains ranged from 80 to 92. Overall, these results show that the adapted CPG achieved a satisfactory level of validity, indicating its high-quality and applicability.

**Fig. 2:**
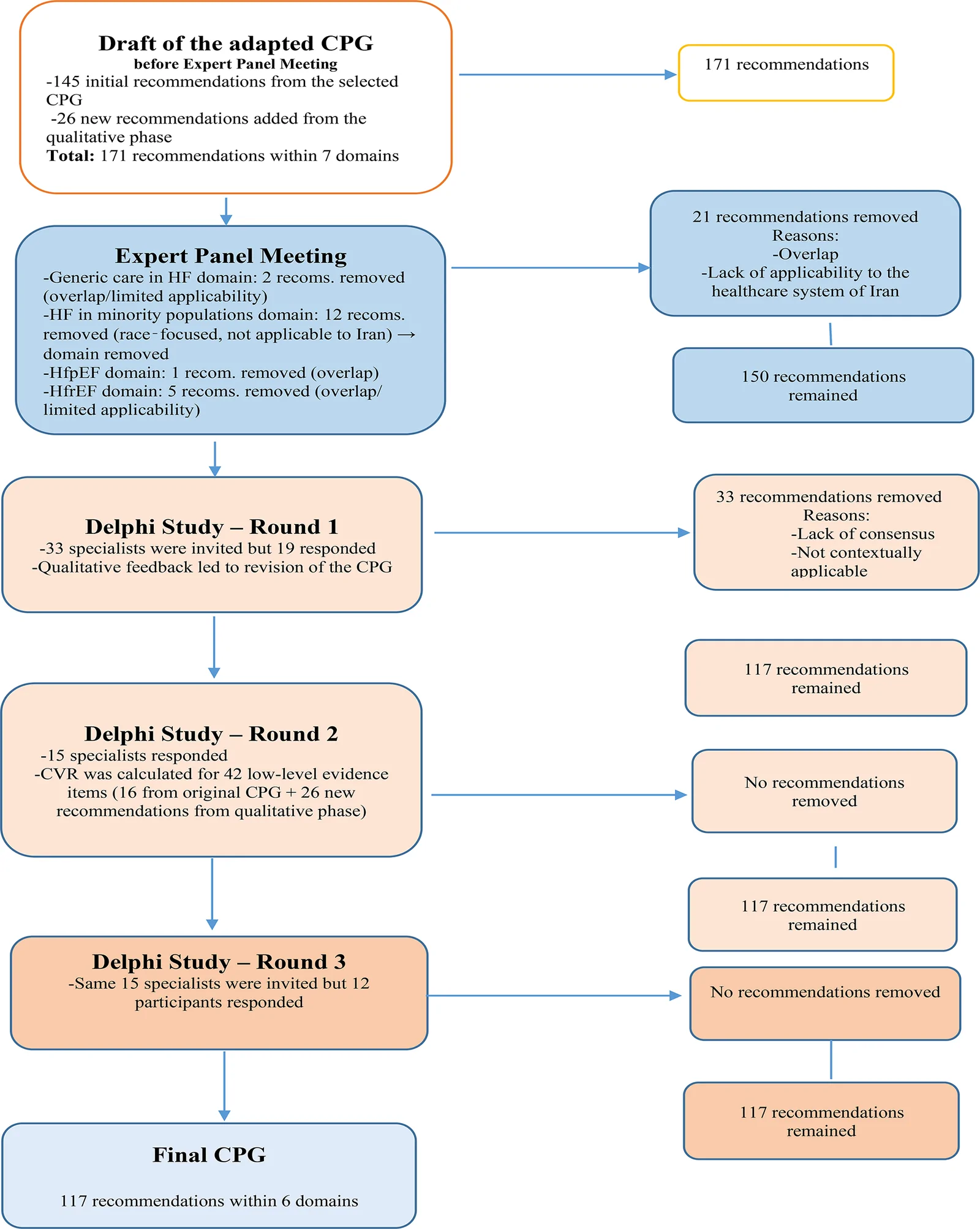
Flow diagram of recommendation selection during the expert panel meeting and three rounds of Delphi. HF: Heart Failure; HFrEF: Heart Failure with Reduced Ejection Fraction; HfpEF: Heart Failure with Preserved Ejection Fraction; CPG: Clinical Practice Guideline

The flow diagram of the selection of CPG recommendations during the expert panel meeting and three Delphi rounds is shown in Fig. 2.

### Results of the finalization phase (Steps 8–10)

**Fig. 3:**
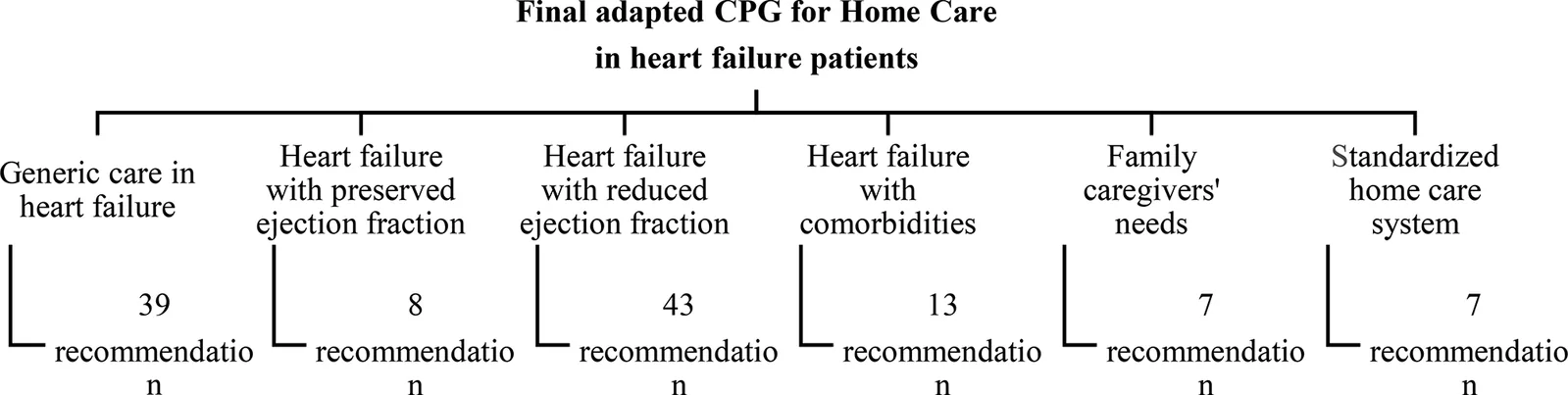
Diagram of the six domains of the final adapted version of the Clinical Practice Guideline (CPG) for home-based nursing care in adult patients with heart failure

In this phase, the final version of the adapted CPG for home care of heart failure patients was produced, comprising 117 recommendations across six domains: “Generic heart failure,” “Heart failure with preserved ejection fraction,” “Heart failure with reduced ejection fraction,” “Heart failure with comorbidities” (four old domains), “Family caregivers’ needs” and “Standardized home care system” (two new domains) (Fig. 3).

The details of the adapted CPG recommendations with levels of evidence and a list of medications for heart failure patients are provided in the Supplementary Files 3–5.

The final draft of this adapted CPG was submitted to the Ministry of Health and Medical Education of Iran for official approval and implementation.

## Discussion

The transition of patients’ care from hospital to community has increased the need for greater investment in delivering healthcare services at home. Implementing home healthcare programs for patients with chronic conditions in the current healthcare system of Iran is essential to prevent readmission and improve patients’ Quality of Life [32].

Evidence suggests that the expansion of CPGs, supported by scientific documentation in line with international standards, can provide the foundation for the delivery of consistent, coordinated, and high-quality home healthcare for patients with chronic diseases, including heart failure, nationwide [10]. Thus, this study was conducted to select and adapt the “Adapting Heart Failure Guidelines for Nursing Care in Home Health Settings,” as a robust CPG with general and home healthcare specific recommendations [31] for delivering healthcare services at home for adult patients with heart failure in the healthcare system of Iran. To expand the theoretical framework of our study and justifying this adaptation, we selected the 10-step framework of the PGEAC for guideline adaptation [24] in order to move beyond a simple “cultural adjustment” and contemplate the structural friction between this international Guideline and the contextual realism of the healthcare system of Iran. Therefore, this study was conducted to produce a robust and friendly CPG version, taking into account the cultural and structural characteristics of the healthcare system and the specific needs of Iranian patients with heart failure, in order to improve the quality of home healthcare and patient health outcomes.

From our study results an adapted version of the CPG for delivering healthcare services at home for adult Iranian patients with heart failure was produced. It comprises 117 recommendations within six main domains, consisting of “Generic care in heart failure,” “Heart failure with preserved ejection fraction,” “Heart failure with reduced ejectionfraction,” “Heart failure with comorbidities,” “Family caregivers’ needs” and “Standardized home care system.” Our findings showed that a substantial proportion of the recommendations in the original guideline were not directly transferable to Iran’s home healthcare system without contextual assessment. During the expert panel meeting phase, 21 recommendations were removed due to misalignment with the country’s healthcare system, available resources, and defined professional roles. Furthermore, the complete removal of the ‘Heart Failure in Minority Populations’ domain with 12 recommendations exemplified this lack of fit, as its recommendations were based on specific racial considerations that were not relevant to the Iranian population. Also, in the first round of the Delphi process, an additional 33 recommendations were excluded.

Interestingly, the qualitative findings identified two additional areas; “Family caregivers’ needs” and “Standardized home care system,” which led to the development of 26 new recommendations. These findings highlight that guideline adaptation is a necessary prerequisite for generating contextually appropriate and implementable recommendations across different healthcare settings.

The initial results from the step of systematic review of CPGs indicated that there are 10 relevant CPGs in the field of home healthcare for adult patients with heart failure. Evaluation of these CPGs revealed that eight are general guidelines for heart failure patients [18, 33–39], while two are specialized home care guidelines [37, 40]. Evidence suggests that general heart failure guidelines can also be applied in home healthcare, although their focus is primarily on hospital and clinical settings, and have only limited references to home-based healthcare services. In our study in the process of selecting the best CPG, all general guidelines were excluded based on evaluation by the AGREE-II, and only two specialized home care guidelines were remained.

The first home care guideline; “Practical Guide on Home Health in HF Patients,” represents the first serious attempt to address home healthcare for patients with heart failure and was produced in Sweden in 2013 [40]. Its purpose was to describe the characteristics of home healthcare for heart failure patients and provide guidance for delivering home healthcare by healthcare providers [40]. The second home care guideline; ”Adapting HF Guideline for Nursing Care in Home Health Settings,” was developed in the United States in 2014 [31]. This guideline adapted general nursing care recommendations for home healthcare to fit the scope and expectations of nursing practice in home healthcare settings. It includes 172 recommendations across five domains: “Generic care in heart failure,” “Heart failure in special and minority populations,” “Heart failure with preserved ejection fraction,” “Heart failure with reduced ejection fraction,” and “Heart failure with comorbidities” [31]. A review of specialized home healthcare guidelines in details showed that the ”Practical Guide on Home Health in Heart Failure Patients” (2013) was developed based on a literature review, international experiences, evaluation of heart failure management programs, expert opinions, and clinical trial data. This guideline presents a comprehensive framework highlighting key aspects of home healthcare for heart failure patients, including multidisciplinary care, patient and family engagement, the design of targeted care plans, patient education, promotion of self-care, access to care, and treatment optimization [40]. However, despite providing a comprehensive framework, this guideline has significant practical limitations that confine its applicability in the healthcare system of Iran. First, its primary focus was on macro-level components and the overall structure of care, with most recommendations remaining at a conceptual level and lacking operational details for nurses in real home settings. Second, it did not address the specialized aspects of nursing care for heart failure patients, also it did not provide specific strategies for implementing care within the home environment. Therefore, while this guideline serves as a valuable conceptual framework, it could not independently function as a comprehensive and practical guideline suitable for the context of the healthcare system in Iran. Thus, based on the AGREE-II, the second specialized home healthcare guideline; “Adapting Heart Failure Guidelines for Nursing Care in Home Health Settings” (2014), was selected as the best and preferred guideline for adaptation in our study. Evidence shows that its recommendations were extracted from established international guidelines, such as those from the AHA and HFSA, and adjusted to meet the specific needs of patients during home healthcare nursing. The use of the Delphi method further enhanced the credibility and applicability of these recommendations, and the categorization based on different patient groups (e.g., cardiac function characteristics or heart failure patients with comorbidities) provided additional flexibility with a stronger focus on the role of nurses in patient care [31]. However, this CPG and application of it have also limitations, for example some recommendations remained general, certain interventions required advanced clinical tools or data not readily accessible in the home setting, and parts of the interventions exceeded the legal scope of practice for nurses in the healthcare system of Iran.

Therefore, although the second specialized home healthcare guideline represented an important step toward enhancing home healthcare for patients with heart failure, gaps remained in providing a comprehensive framework for countries with different healthcare systems, such as Iran. In the present study, a multi-method design was used to integrate valid international evidence from the selected CPG; ”Adapting HF Guideline for Nursing Care in Home Health Settings” with local findings. As a result, an integrated CPG tailored to the needs of heart failure patients and their family caregivers at home was produced for use in the healthcare system of Iran. This adapted CPG includes 117 recommendations, including 26 new recommendations derived from the qualitative phase, organized into six main domains. Four domains were old and adapted from the selected guideline; namely “Generic care in heart failure,” “Heart failure with preserved ejection fraction,” “Heart failure with reduced ejection fraction,” and “Heart failure with comorbidities” [31]. Furthermore, two innovative domains; “Family caregivers’ needs” and “Standardized home care system,” were derived from the qualitative findings of the study, and added to the existing domains. This feature distinguishes the present adapted CPG from other existing CPGs.

A review of the new domains reveals that the first new domain; “Family caregivers’ needs,” acknowledges caregivers as a cornerstone of support who play a critical role in improving patient health. Their physical and psychological well-being directly affects the quality of care and patient outcomes [41]. Home care teams, by identifying and addressing the needs of family caregivers and leveraging their capacities, can simultaneously support this group and improve the quality of care for patients with heart failure.

Additionally, the second new domain; “Standardized home care system,” emphasizes the importance of providing a safe and structured environment for patients. Current home care teams often lack sufficient training and resources to assess and improve the infrastructure of patients’ homes. Comprehensive planning is essential to manage adverse events, such as emergency care during crises, fall prevention, indoor air quality improvement, safety of sanitary facilities, and enhancing patients’ ability to perform daily activities [42]. Moreover, the participation of family members in organizing and maintaining the safety of the home environment plays a crucial role in ensuring the quality of patient care.

It is noteworthy that the adapted CPG in this study, offers several advantages over existing guidelines. It places special emphasis on family caregiver needs, standardizes the home care system, aligns recommendations with the cultural context and healthcare structure of Iran, and provides practical applicability for nurses and other healthcare providers, including general practitioners and psychologists. Since this adapted CPG was developed with active involvement of stakeholders, including patients, families, and healthcare providers, this approach may enhance acceptance of recommendations and promote more effective clinical implementation [43]. Furthermore, the systematic review of scientific records, extraction of recommendations from credible sources, and classification of recommendations based on the level of evidence, have strengthened the scientific credibility of the CPG [44]. In addition, the validation process of the adapted CPG, conducted through a three-round Delphi method, enhanced its feasibility, scientific rigor, and operational applicability.

Overall, from the results of this study, the adapted version of the home care guideline for patients with heart failure was produced for use in the healthcare system of Iran. It can serve as a key guide to improve the quality of home healthcare services, reduce healthcare costs, and decrease hospital readmissions. Future research is recommended to evaluate the effectiveness of this adapted CPG in terms of clinical outcomes and cost-effectiveness through interventional, comparative, and longitudinal studies.

### Implications of the study

The adapted guideline can help provide standard nursing care for patients with heart failure at home and community settings. During use of this adapted guideline, through increasing patient education, symptom monitoring, and the active involvement of family caregivers, patient self-care may be improved and potentially reduce hospital readmissions. However, its effective implementation may face several challenges at the clinical and community levels, including a shortage of nursing staff in home healthcare services, high workload, limited outpatient and home healthcare resources, the need for targeted training for nurses, and the absence of online platform for home healthcare, systematic mechanisms to monitor and evaluate the implementation of the adapted guideline.

From a health policy perspective, the findings of this study highlight the need to develop home healthcare standards, strengthen support for family caregivers, and design evidence-based healthcare service packages for heart failure management at home. It also underscore the importance of expanding home healthcare services and integrating these services into the PHC network, and involving nurses, physicians, and other healthcare providers in the network and developing specific programs for management of patients with heart failure at home.

### Strengths and limitations of the study

One of the strengths of this study is the integration of a multi-method study design within the steps of the PGEAC and the development of a comprehensive, specialized CPG for home healthcare of patients with heart failure. The adapted CPG is specifically tailored to the structure of the national healthcare system of Iran and the needs of patients and their families in this system. Given its structured and evidence-based approach, this CPG has significant potential for application in other countries with similar healthcare systems. Beyond providing a practical framework for designing educational programs for nurses and other healthcare providers for home healthcare, the adapted CPG may theoretically contribute to reducing healthcare costs, decreasing hospital readmissions, and improving the Quality of Life of patients with heart failure and their families. Additionally, its content can serve as a foundation for developing national standards, innovative implementation models, healthcare service packages and integrating home healthcare services into the healthcare system of the country.

However, several limitations should be considered when interpreting the findings. First, since the adaptation process was conducted within the context of a specific healthcare system, its generalizability to countries with different healthcare systems could be limited. During the systematic review search of CPGs, access to certain guidelines was also limited; to remove this limitation, correspondence with the responsible authors was conducted through email by the first (L.H) and last (C.R) authors, and the required guidelines were obtained after getting permission from the developers. Additionally, in the Delphi study, to ensure continuous participation of specialists throughout the three Delphi rounds, active engagement was promoted through effective communication, sending three reminder emails, and ensuring that participants received the final results after completing the study.

## Conclusion

This multi-method study was conducted within the framework of the 10-step PGEAC to adapt the “Adapting Heart Failure Guidelines for Nursing Care in Home Health Settings,” as a specialized CPG for home-based nursing care of patients with heart failure in Iran. The adapted version of the CPG with 117 evidence-based recommendations in six domains was produced based on the structural and operational characteristics of the healthcare system and the needs of patients and families in our context. The adapted CPG can serve as a practical tool for both general and specialist nurses, as well as other healthcare professionals such as physicians in the healthcare system of Iran. It provides a framework to enhance the quality of home healthcare services, improve the Quality of Life of adult patients with heart failure and their families, and standardize home care practices nationwide.

Future steps should include formal endorsement of the adapted CPG by policymakers in the healthcare system of the country, and its integration into official home care programs for patients with heart failure at the provincial level. Evaluation of its cost-effectiveness and feasibility in real-world settings by researchers will help identify strengths and areas to improve in future versions. Thus, this adapted version of the CPG can not only address the needs of patients and their families, but can also contribute to the global discourse on advancing home care models.

## Supplementary Information

Below is the link to the electronic supplementary material.

Supplementary Material 1

## Data Availability

The interview dataset generated and analysed during the current study are not publicly available due to the anonymity and confidentiality of participants’ information. However, on a reasonable request the data could be available from the corresponding authors.

## Abbreviations

ACC: American College of Cardiology
AHA: American Heart Association
AGREE-II: Appraisal of Guidelines for Research and Evaluation II
CPG: Clinical Practice Guideline
HFSA: Heart Failure Society of America
NYHA: New York Heart Association
