# Supplementary material for: Adaptation of a clinical practice guideline for home care nursing in heart failure patients: a multi-method study
Source: BMC Nurs. 2026 Jul 4;25:887. doi: 10.1186/s12912-026-04774-x (PMC13613711; doi:10.1186/s12912-026-04774-x)
Supplement: Supplementary file 1 [file 12912_2026_4774_MOESM1_ESM.docx]

**Supplementary File 1**

**Table 1.** Grading of Recommendations Based on Evidence (According to the Heart Failure Society of America: HFSA Guidelines)

| **Recommendation Grade** | **Levels of Evidence** | **Description** |
| --- | --- | --- |
| A (Strong Evidence) | Systematic reviews, meta analyses, multicenter clinical trials, randomized controlled trials | High quality evidence with consistent results |
| B (Moderate Evidence) | Cohort and case–control studies, meta analyses of observational studies | Moderate quality evidence with reasonably consistent results |
| C (Weak Evidence) | Expert opinions, observational studies | Limited evidence or based on expert consensus |
| Amiodarone | Photosensitivity, nausea, thyroid dysfunction, sleep disturbances, corneal microdeposits | Hepatic or pulmonary fibrosis, thyroid storm, pro arrhythmia |
| Inotropes | Nausea, abdominal pain | Arrhythmia, cardiotoxicity |

Note

The adapted clinical practice guideline has been categorized into two major domains:

1. What nurses should perform
2. What nurses should know

Some recommendations are classified under “what nurses should know” because in Iran nurses do not have legal authorization to prescribe medications. However, they should be well informed about pharmacological management in heart failure patients including medications that are contraindicated. For instance, nonsteroidal anti inflammatory drugs (NSAIDs), including cyclooxygenase inhibitors, are not recommended in patients with chronic heart failure

**Supplementary File 2**

**INTERVIEW GUIDE**

**Examples of interview questions**

**Interview with Patients:**

1. How do you see your needs if you were to be cared for at home?

2. How are your needs met at home?

3. What needs for home care did you have that were not met despite your expectations?

**Interview with Family Caregivers:**

1. Please describe a day in your life caring for your patient at home.

2. What are the challenges you face while caring for your patient?

3. What did you do/do you do to solve the problem?

**Interview with Different Healthcare Providers and Specialists:**

1. Based on your experience, what are the needs of the family and patients at home?

2. What is the role of nurses in home care for patients with heart failure?

3. What types of social supports can help patients with heart failure?

4. Under what circumstances and how should patients with heart failure be referred for home care?

5. Could you please explain what should be considered when assessing and taking a history of a patient with heart failure?

6. Please explain the laboratory evaluation of patients with heart failure and please share your experience in this regard.

7. What special attention and care do elderly patients with heart failure need?

8. Explain the nutrition of patients with heart failure and please share your experience in this regard.

9. How much should sodium and fluid restriction be in patients with heart failure?

10. What is the fluid restriction in patients taking diuretics?

11. Please explain the use of steroids and nutritional supplements for cachexia and share your experience?

12. Please explain the medications needed by patients with heart failure?

13. Are calcium channel blockers used in patients with heart failure who need ventricular rate control and are intolerant to beta-blockers? Please share your experience in this regard.

14. Is beta-blocker treatment recommended in patients with worsening heart failure symptoms, for example, patients with fluid overload or frequent hypoglycemia, or those with asthma? Please share your experience in this area.

15. Is high-dose digoxin recommended in patients with heart failure to control heart rate? How? Please share your experience in this area.

16. Is warfarin recommended for all patients with heart failure with a history of systemic or pulmonary embolism? How? Please share your experience in this area.

17. Do you recommend long-term use of a positive inotropic drug such as milrinone for patients with heart failure and reduced left ventricular ejection fraction? Please share your experience in this area.

18. Should patients with heart failure be treated for chronic stable angina? Please share your experience in this area.

Furthermore, the researcher used probing questions such as “Could you please explain more?” and “Could you please give an example?” to encourage participants and obtain more in-depth information.

**Supplementary File 3**.

**Table 2.** Recommendations of the Adapted Clinical Practice Guideline for Home Care Nursing in Heart Failure Patients with levels of evidence

| **Levels of Evidence (Reference)** | **Guideline Recommendations for Generic Heart Failure (HF)**  **(These recommendations should be implemented by nurses)** |
| --- | --- |
| **C**  **(1)** | 1. Nurses should obtain a complete medical history and perform a thorough physical examination for all patients with heart failure (HF). |
| **C**  **(1)** | 2. The clinical severity of HF should be assessed through detailed history and physical examination. Reversible causes of HF should be identified, and evaluations for coronary artery disease, myocardial ischemia, life threatening arrhythmias, exacerbating factors, adverse effects of therapy, and barriers to adherence should be conducted. |
| **B**  **(2)** | 3. HF symptoms should be comprehensively evaluated, including dyspnea, fatigue, exercise intolerance, and fluid retention, as well as atypical signs such as anginal pain, thromboembolic events, sleep disturbances, palpitations, and episodes of syncope or presyncope. |
| **A**  **(3)** | 4. The patient’s functional capacity should be evaluated and documented using standardized tools such as the New York Heart Association (NYHA) classification. |
| **C**  **(4)** | 5. The patient’s volume status should be assessed through clinical findings such as paroxysmal nocturnal dyspnea, orthopnea, weight changes, vital signs, pulmonary rales, S3 gallop, jugular venous distention, hepatomegaly, hepatojugular reflux, peripheral edema, and ascites. |
| **B**  **(5)** | 6. Blood pressure control is recommended for all patients with HF. |
| **B**  **(3, 6)** | 7. At each follow-up visit, nurses should assess functional capacity, activity level, body weight changes, patient understanding of the disease, adherence to sodium restriction and medications, arrhythmia history, and presence of new or worsening comorbidities. |
| **C**  **(7, 9)** | 8. Dietary sodium restriction (2–3 g/day) is recommended for all HF patients, with stricter limits (≤2 g/day) in moderate to severe HF. Fluid restriction (<2 L/day) should be considered in patients with severe hyponatremia (serum sodium <130 mEq/L) or persistent fluid retention despite optimal diuretic therapy. |
| **B**  **(10, 11)** | 9. Nutritional assessment is recommended for patients with advanced HF and cardiac cachexia, including evaluation of nitrogen balance, caloric intake, and prealbumin levels, to guide nutritional supplementation. |
| **B**  **(12-14)** | 10. Sleep disturbances, restless leg syndrome, and depression should be screened and treated appropriately in patients with HF. Non–habit-forming medications are preferred for sleep induction. |
| **B**  **(15)** | 11. Nonpharmacological stress reduction techniques should be identified and encouraged to help reduce anxiety in patients with HF. |
| **B**  **(16-18)** | 12. Annual influenza and pneumococcal vaccinations are recommended for all patients with HF, unless contraindicated. |
| **B**  **(19)** | 13. Evaluate patients’ physical and mental capacity for returning to work or initiating new employment, taking into account functional status and occupational demands. |
| **B**  **(20-22)** | 14. Exercise testing should be considered to determine safe activity levels. Supervised exercise programs of moderate intensity (30 minutes, 5 days per week) are recommended, including proper warm up and cool down sessions. |
| **B**  **(23)** | 15. Hypoxemia at rest or during exercise should be evaluated. Supplemental oxygen is not routinely recommended unless a coexisting pulmonary disorder is present. |
| **B**  **(24, 25)** | 16. Screening for chronic depression is recommended in patients with HF. |
| **B**  **(7)** | 17. Patients and caregivers should be educated to recognize early signs of fluid retention and to implement prompt self-management strategies. |
| **C**  **(26)** | 18. Selected patients should be educated on self-adjustment of diuretic dose in response to early weight gain (typically 1–2 kg over a few days) due to fluid overload. |
| **A** | 19. Individualized care should be provided for elderly HF patients, considering altered drug metabolism and tolerance. |
| **A**  **(27)** | 20. Discussions regarding prognosis and quality of life should be initiated with patients and families. End-of-life preferences should be addressed early while the patient can still participate in decision-making. Spiritual assessment by a trained chaplain may be beneficial when needed. |
| **A**  **(27)** | 21. Patients and families should receive education on palliative and hospice care options. Discussions regarding resuscitation preferences and emergency care planning are recommended, with referrals to trained spiritual or ethics counselors as appropriate. |
| **A**  **(15, 27)** | 22. Patients with advanced HF and their families should be informed that, despite advanced therapies, the risk of mortality remains high. End-of-life care should be offered to patients with poor quality of life and limited daily functioning, with access to trained spiritual counselors when possible. |
| **A**  **(28, 29)** | 23. Coordination with the patient’s physician is recommended for individualized end-of-life management plans, including continuation of essential HF medications, effective symptom control, palliative measures, and avoidance of unnecessary testing. Psychological and spiritual support from multidisciplinary teams should be provided when indicated. |
|  | **Recommendations 24, 25, 26, and 27 should be known by nurses ( not performed by them)** |
| **B**  **(30)** | 24. Daily weight monitoring is recommended for all patients with HF to detect early fluid retention. |
| **B**  **(30)** | 25. Patients should be instructed to record and report any rapid weight gain (≥2 kg over 3 days) to the healthcare team. |
| **C**  **(26)** | 26. Peripheral edema and ascites should be assessed at each visit and documented for trend analysis. |
| **B**  **(31, 32)** | 27. Monitoring of blood pressure, heart rate, respiratory rate, and oxygen saturation should be performed routinely in all HF patients. |
| **C** | 28. Laboratory assessments, including electrolytes, renal function, liver function, and complete blood count, should be performed regularly based on clinical status and medication use. |
| **C** | 29. Medication adherence should be assessed, and barriers to adherence should be identified and addressed in collaboration with patients and caregivers. |
| **C** | 30. Patients should receive education regarding the purpose, dosage, and potential side effects of all prescribed HF medications, including diuretics, ACE inhibitors, ARBs, beta blockers, aldosterone antagonists, digoxin, and anticoagulants. |
| **C** | 31. Caregivers should be educated to assist with medication administration and monitoring for adverse effects. |
| **C** | 32. Patients and caregivers should receive training on recognizing signs of worsening HF, including dyspnea, fatigue, orthopnea, paroxysmal nocturnal dyspnea, and increased edema. |
| **C** | 33. Symptom diaries or home monitoring tools should be encouraged to facilitate early detection of decompensation. |
| **C** | 34. Patients with comorbid conditions (e.g., diabetes, hypertension, COPD) should have integrated care plans that address all chronic diseases. |
| **C** | 35. Education regarding lifestyle modification, including low sodium diet, fluid management, smoking cessation, weight management, and safe physical activity, is recommended. |
| **C** | 36. Psychological support and counseling should be provided to address anxiety, depression, or social isolation in HF patients. |
| **C** | 37. Referral to multidisciplinary HF clinics or home care services should be considered to optimize outcomes and provide ongoing support. |
| **C** | 38. Regular evaluation of functional status and exercise tolerance should guide individualized exercise programs and rehabilitation. |
| **C** | 39. Patients and caregivers should be actively involved in shared decision-making regarding treatment options, hospitalizations, and end-of-life care planning |

| **Level of Evidence** | **Recommendations for HF with normal EF**  **(These recommendations should be understood by nurses)** |
| --- | --- |
| **B**  **(33)** | 1. Diuretic therapy is recommended for all patients with heart failure who have clinical evidence of fluid overload, including those with preserved left ventricular ejection fraction (LVEF). Treatment may be initiated with a thiazide or loop diuretic. |
| **С**  **(34)** | 2. Angiotensin converting enzyme (ACE) inhibitors should be considered in all patients with HF and preserved LVEF who have symptomatic cardiovascular disease, diabetes, or additional risk factors. For patients who are intolerant to ACE inhibitors (e.g., due to cough), angiotensin receptor blockers (ARBs) should be considered. |
| **A**  **(35)** | 3. Beta blocker therapy is recommended for patients with HF and preserved LVEF who have a history of myocardial infarction. |
| **B**  **(36)** | 4. Beta blockers are also recommended for patients with HF and preserved LVEF who have hypertension. |
| **B**  **(37)** | 5. Beta blocker therapy is recommended for patients with HF and preserved LVEF who have atrial fibrillation requiring control of ventricular rate. |
| **C**  **(26)** | 6. Calcium channel blockers (verapamil or diltiazem) should be considered for patients with HF and preserved LVEF who have atrial fibrillation and are intolerant to beta blockers, for ventricular rate control. |
| **A**  **(38)** | 7. Calcium channel blockers may also be considered in patients with HF and preserved LVEF who experience mild angina symptoms. |
| **B**  **(39)** | 8. Calcium channel blockers should be considered for patients with HF and preserved LVEF who have hypertension. |
| **Level of Evidence** | **Recommendations for HF with co morbidities**  **(These recommendations should be known and implemented by nurses)** |
| **A**  **(40)**  **)** | 1. It is recommended that blood pressure be managed appropriately to reduce both systolic and diastolic levels. More than one medication may be required. If tolerated, the resting blood pressure should be maintained below 130/80 mmHg. |
| **B**  **(41)** | 2. If blood pressure remains above 130/80 mmHg, it is recommended to add a thiazide diuretic in addition to a dihydropyridine calcium channel blocker or other antihypertensive agents. |
| **A**  **(42)** | 3. Antiplatelet therapy with aspirin is recommended to reduce vascular events in patients with heart failure and coronary artery disease, unless contraindicated. |
| **C**  **(43)** | 4. Antiplatelet therapy with clopidogrel is also recommended to reduce vascular events in patients with heart failure and coronary artery disease, unless contraindicated. |
| **B**  **(43)** | 5. Clinicians should prescribe antiplatelet agents to prevent myocardial infarction and death in patients with heart failure who have underlying coronary artery disease. |
| **B**  **(44)** | 6. For the treatment of angina in patients with heart failure, clinicians should use nitrates and beta blockers. |
| **B**  **(45)** | 7. Calcium channel blockers (CCBs) may be considered for patients with heart failure who continue to experience angina despite optimal use of beta blockers and nitrates.   o Amlodipine and felodipine are the preferred calcium channel blockers in patients with angina and reduced systolic function.  o Based on current evidence, first generation CCBs (e.g., diltiazem, verapamil) should be avoided in patients with coronary artery disease, heart failure, and LVEF < 40%, unless required for rate control or other specific indications. |
| **A**  **(46)** | 8. Clinicians should prescribe anticoagulant therapy for patients with heart failure who have paroxysmal or persistent atrial fibrillation or a previous thromboembolic event. |
| **A**  **(47)** | 9. Clinicians should control ventricular rate in patients with heart failure and atrial fibrillation using a beta blocker (or amiodarone if beta blockers are contraindicated or not tolerated). |
| **A**  **(48)** | 10. Digoxin administration is reasonable for rate control in patients with heart failure and atrial fibrillation. |
| **A**  **(49)** | 11. Amiodarone therapy is reasonable to reduce recurrence of atrial arrhythmias and ventricular arrhythmias associated with ICD discharges. |
| **A**  **(49)** | 12. Class I or III antiarrhythmic drugs are not recommended for the prevention of ventricular arrhythmias in patients with heart failure. |
| **A**  **(50)** | 13. The use of antiarrhythmic drugs as primary therapy for asymptomatic ventricular arrhythmias or for survival improvement in patients with heart failure is not indicated. |

| **Level of Evidence** | **Recommendations in the domain of heart failure with HF with Red EF**  **(These recommendations should be known by nurses)** |
| --- | --- |
| **A**  **(51)** | 1. The use of angiotensin converting enzyme (ACE) inhibitors is recommended as routine therapy for both symptomatic and asymptomatic patients with left ventricular ejection fraction (LVEF) <40%, unless contraindicated. |
| **A**  **(52)** | 2. In patients who develop cough due to ACE inhibitor therapy, substitution with an angiotensin receptor blocker (ARB) is recommended. ARBs should be avoided in cases of hyperkalemia (serum potassium >5.5 mmol/L) or acute renal failure. |
| **B**  **(53, 54)**  **(61, 62)** | 3. In patients who develop angioedema from ACE inhibitors, ARBs may be cautiously substituted due to their lower risk of causing angioedema. |
| **B**  **(55)**  **(63)** | 4. Patients who are intolerant to ACE inhibitors due to hyperkalemia or acute renal dysfunction are likely to experience similar adverse effects with ARBs. In such cases, the combination of oral hydralazine and nitrate should be considered as an alternative. |
| **A**  **(56)** | 5. ARBs may be considered as first line therapy instead of ACE inhibitors in patients with heart failure following myocardial infarction (MI). |
| **B**  **(57, 58)** | 6. ARBs may also be considered as primary therapy in patients with chronic heart failure and reduced LVEF when ACE inhibitors are not tolerated. |
| **A**  **(58)** | 7. ARBs are considered equivalent to ACE inhibitors in post MI patients with heart failure and reduced LVEF. |
| **A**  **(58)** | 8. The concomitant use of ACE inhibitors, ARBs, and beta blockers is not recommended in patients with recent acute MI and reduced LVEF due to increased risk of adverse effects. |
| **A**  **(59)** | 9. Beta blockers with proven efficacy in clinical trials are recommended for all patients with LVEF <40%. |
| **A**  **(60)** | 10. One of the three evidence based beta blockers — bisoprolol, carvedilol, or metoprolol succinate — is recommended for all stable patients with current or prior symptoms of HF and reduced LVEF, unless contraindicated. |
| **B**  **(60)** | 11. A combination of a beta blocker and an ACE inhibitor (or ARB) is recommended as routine therapy for asymptomatic post MI patients with HF and LVEF <40%. |
| **C** | 12. Beta blocker therapy should be used in patients with diabetes with recurrent hypoglycemia, ischemia, or asthma, with caution. In patients with marked bradycardia (<55 bpm) or significant hypotension (systolic BP <80 mmHg), beta blockers should be used with great caution. |
| **B**  **(60)** | 13. Beta blocker therapy is recommended in most patients with HF and reduced LVEF, even when comorbid conditions such as diabetes, chronic obstructive pulmonary disease (COPD), or peripheral vascular disease are present. |
| **C**  **(59)** | 14. It is recommended that beta blocker therapy be continued in most patients who experience worsening HF symptoms during chronic treatment, unless they develop cardiogenic shock, severe fluid overload, or symptomatic bradycardia. |
| **A**  **(61)** | 15. Aldosterone antagonist therapy is recommended for patients with New York Heart Association (NYHA) class IV heart failure and LVEF <35% who are already receiving standard therapy including diuretics. |
| **A**  **(62)** | 16. Aldosterone antagonist therapy should also be considered in patients with acute MI who have clinical signs or a history of HF, diabetes mellitus, and LVEF <40%. Such patients should already be receiving standard therapy, including an ACE inhibitor (or ARB) and a beta blocker. |
| **A**  **(62)** | 17. Aldosterone antagonists are not recommended in patients with serum creatinine >2.5 mg/dL (or creatinine clearance <30 mL/min), serum potassium >5.0 mmol/L, or when used concurrently with potassium sparing agents. |
| **A**  **(63)** | 18. Serum potassium levels should be monitored after initiating or adjusting the dose of an aldosterone antagonist. |
| **A**  **(64)** | 19. Potassium supplementation is not recommended in patients receiving aldosterone antagonists unless resistant hypokalemia (serum K⁺ <4.0 mmol/L) is present. |
| **B**  **(65)** | 20. The addition of oral hydralazine and nitrate therapy is reasonable for patients with reduced LVEF who remain symptomatic despite optimal treatment with ACE inhibitors and beta blockers. |
| **B**  **(33)** | 21. The initial dose of diuretics may be increased as needed to relieve congestion. Multiple dose adjustments over several days or, in some cases, weeks may be required in patients with marked fluid overload, as evidenced by edema or ascites. Once an effective diuretic response is achieved with short acting loop diuretics, dividing the total daily dose into two or three administrations enhances diuresis with less physiological disturbance compared with larger single doses. |
| **A**  **(66)** | 22. Oral torsemide may be considered in patients with poor gastrointestinal absorption or variable diuretic response, particularly in those with right sided heart failure and fluid retention despite high dose therapy with other loop diuretics. |
| **A**  **(33)** | 23. Intravenous administration of loop diuretics may be required to achieve adequate relief of congestion. |
| **B**  **(67)** | 24. Careful monitoring for adverse effects—including electrolyte imbalance, symptomatic hypotension, renal dysfunction, or worsening renal function—is recommended in patients treated with diuretics, especially when high doses or combinations are used. Routine laboratory testing and clinical assessments should guide dose titration based on clinical response. |
| **B**  **(68)** | 25. Digoxin may be considered to improve symptoms in patients with HFrEF (LVEF <40%) who remain symptomatic (NYHA class II–IV) despite optimal standard therapy, including ACE inhibitors and beta blockers. |
| **B**  **(69)** | 26. Dose adjustment of digoxin should be based on body mass, renal function, and concomitant medications. In most patients, a daily dose of 0.125 mg is appropriate, with target serum concentrations <1.0 ng/mL, typically 0.7–0.9 ng/mL. |
| **A**  **(70)** | 27. Oral anticoagulation with warfarin (target INR 2.0–3.0) is recommended for all patients with HFrEF and chronic, persistent, or permanent atrial fibrillation, unless contraindicated. |
| **C**  **(70)** | 28. Warfarin therapy (INR 2.0–3.0) is also recommended for patients with a history of systemic or pulmonary embolism, including stroke or transient ischemic attack, unless contraindicated. |
| **B**  **(71)** | 29. Long term antiplatelet therapy, typically with low dose aspirin (75–81 mg daily), is recommended for patients with ischemic cardiomyopathy, regardless of concurrent ACE inhibitor therapy. |
| **B**  **(71)** | 30. Warfarin (INR 2.0–3.0) or clopidogrel (75 mg daily) may be considered as alternatives to aspirin for secondary prevention of vascular events after myocardial infarction. |
| **B**  **(72)** | 31. Routine aspirin use is not recommended in patients with heart failure in the absence of atherosclerotic vascular disease. |
| **B**  **(44)** | 32. Long term anticoagulation should be considered in patients with ischemic or nonischemic cardiomyopathy and left ventricular thrombus, depending on thrombus characteristics such as size, mobility, and calcification. |
| **A**  **(73)** | 33. Antiarrhythmic drugs, including amiodarone, are not recommended for the primary prevention of sudden cardiac death in patients with HFrEF. |
| **A**  **(74, 75)** | 34. When initiating amiodarone, potential drug interactions should be reviewed. Maintenance doses of digoxin, warfarin, and certain statins should be reduced and closely monitored. Laboratory assessment of drug activity or serum concentrations following amiodarone initiation is recommended. |
| **A**  **(76)** | 35. The triple combination of an ACE inhibitor, an ARB, and an aldosterone antagonist is not recommended due to a high risk of hyperkalemia. |
| **A**  **(77)** | 36. Addition of an ARB or aldosterone antagonist may be considered in patients with severe heart failure or post–myocardial infarction HFrEF who remain symptomatic or worsen despite optimal ACE inhibitor and beta blocker therapy. |
| **A**  **(78)** | 37. In patients with HFrEF and persistent or worsening symptoms despite optimized ACE inhibitor and beta blocker therapy, consideration should be given to additional pharmacologic agents. |
| **B**  **(79)** | 38. A combination of hydralazine and isosorbide dinitrate should be considered in patients with HFrEF who remain symptomatic or deteriorate despite optimal therapy with ACE inhibitors and beta blockers. |
| **B**  **(80)** | 39. Whenever possible, the use of drugs that may adversely affect the clinical status of patients with current or prior HFrEF—such as NSAIDs, most antiarrhythmic agents, and many calcium channel blockers—should be avoided. |
| **A**  **(81)** | 40. Calcium channel blockers are not indicated as routine therapy for patients with current or prior HFrEF. |
| **A**  **(82)** | 41. Nutritional supplements are not indicated as treatment for heart failure in patients with current or prior HFrEF. |
| **B**  **(83, 84)** | 42. Phosphodiesterase type 5 inhibitors (e.g., sildenafil) may be considered in patients with chronic heart failure who experience sexual dysfunction; however, these drugs are contraindicated in patients receiving nitrates. |
| **B**  **(85)** | 43. Nonsteroidal anti-inflammatory drugs (NSAIDs), including COX 2 inhibitors, are not recommended in patients with chronic heart failure, as they significantly increase the risk of renal failure and fluid retention, particularly during ACE inhibitor therapy. |
| **Level of Evidence** | **Recommendations in the domain of family caregivers’ needs**  **(Extracted from the qualitative study to be implemented by nurses)** |
| **C** | 1. Support patients and their families in accessing both formal and informal health care services (psychosocial support through attention and active listening, empathy, and assistance in referring patients to organizations providing mental health, social, and spiritual support). |
| **C** | 2. Based on the care team’s decision, delegate routine care activities to family caregivers in accordance with their physical ability, comprehension, and understanding of care tasks. |
| **C** | 3. Develop a comprehensive home care plan with clear goals for the patient through shared decision making and collaboration with both the patient and the family. |
| **C** | 4. Ask patients and their families about their treatment and care preferences, and support their active participation in the care process. |
| **C** | 5. Home care nurses should provide patients and families with the necessary information regarding disease etiology, prognosis, medications, and treatment based on their needs and level of health literacy. |
| **C** | 6. Home care nurses should inform patients and families about how to contact the home care team when needed. |
| **C** | 7. Home care nurses should provide education on self-care skills for both patients and families (e.g., management of dyspnea, increased heart rate, fatigue, edema, and weight control while using diuretics). |
| **Level of Evidence** | **Recommendations in the domain of standardized home care system**  **(Extracted from the qualitative study to be implemented by nurses)** |
| **C** | 1. Establish effective communication among the multidisciplinary care team, the patient, and the family. |
| **C** | 2. Develop a structured and comprehensive home care plan based on interprofessional collaboration and adherence to standard protocols. |
| **C** | 3. Utilize standardized care protocols and clinical guidelines to optimize patient management at home. |
| **C** | 4. Plan for remote access (e.g., telephone consultations and telehealth support) to enable patients and families to reach rehabilitation and care services and to meet their ongoing needs at home. |
| **C** | 5. Use a combination of different educational media, such as software applications, instructional videos, brochures, and booklets, to provide education for patients and families at home. |
| **C** | 6. Prioritize follow up, continuity of care, and proper documentation of home based care for patients with heart failure. |
| **C** | 7. Ensure that environmental safety standards are maintained in the home setting (e.g., availability of required medical equipment such as oxygen, and proper disposal of infectious waste). |

**References:**

1. Harada R, Mantha Y, Hieda M. Back to Basics: Key Physical Examinations and Theories in Patients with Heart Failure. Heart Fail Clin. 2020 Apr;16(2):139 151. doi: 10.1016/j.hfc.2019.12.001. PMID: 32143759.
2. I Shams P, Malik A, Chhabra L. Heart Failure (Congestive Heart Failure) [Updated 2025 Feb 26]. In: StatPearls [Internet]. Treasure Island (FL): StatPearls Publishing; 2025 Jan . Available from: https://www.ncbi.nlm.nih.gov/books/NBK430873/
3. Clinical Review Report: Dapagliflozin (Forxiga): (AstraZeneca Canada Inc.): Indication: Heart failure with reduced ejection fraction [Internet]. Ottawa (ON): Canadian Agency for Drugs and Technologies in Health; 2021 Mar. Table 12, New York Heart Association Functional Classification. Available from: https://www.ncbi.nlm.nih.gov/books/NBK572289/table/cl4.tab8/
4. Raco J, Peterson B, Muallem S. Assessment of Volume Status in Hospitalized Patients With Chronic Heart Failure. Cardiol Res. 2023 Feb;14(1):2 11. doi: 10.14740/cr1434. Epub 2023 Feb 25. PMID: 36896231; PMCID: PMC9990539.
5. Kitsiou S, Paré G, Jaana M. Effects of home telemonitoring interventions on patients with chronic heart failure: an overview of systematic reviews. J Med Internet Res. 2015;17(3):e63.
6. Dunlay SM, Manemann SM, Chamberlain AM, Cheville AL, Jiang R, Weston SA, et al. Activities of daily living and outcomes in heart failure. Circ Heart Fail. 2015;8(2):261 7.
7. Colin Ramirez E, Sepehrvand N, Rathwell S, Ross H, Escobedo J, Macdonald P, Troughton R, Saldarriaga C, Lanas F, Doughty R, McAlister FA, Ezekowitz JA. Sodium Restriction in Patients With Heart Failure: A Systematic Review and Meta Analysis of Randomized Clinical Trials. Circ Heart Fail. 2023 Jan;16(1):e009879. doi: 10.1161/CIRCHEARTFAILURE.122.009879. Epub 2022 Nov 14. PMID: 36373551.
8. Kato NP, Nagatomo Y, Kawai F, Kitai T, Mizuno A. Fluid Restriction for Patients with Heart Failure: Current Evidence and Future Perspectives. J Pers Med. 2024 Jul 11;14(7):741. doi: 10.3390/jpm14070741. PMID: 39063995; PMCID: PMC11277838.
9. De Vecchis R, Baldi C, Cioppa C, Giasi A, Fusco A. Effects of limiting fluid intake on clinical and laboratory outcomes in patients with heart failure. Results of a meta analysis of randomized controlled trials. Herz. 2016;41(1):63 75.
10. Krysztofiak H, Wleklik M, Migaj J, Dudek M, Uchmanowicz I, Lisiak M, et al. Cardiac Cachexia: A Well Known but Challenging Complication of Heart Failure. Clin Interv Aging. 2020;15:2041 51.
11. Driggin E, Cohen LP, Gallagher D, Karmally W, Maddox T, Hummel SL, Carbone S, Maurer MS. Nutrition Assessment and Dietary Interventions in Heart Failure: JACC Review Topic of the Week. J Am Coll Cardiol. 2022 Apr 26;79(16):1623 1635. doi: 10.1016/j.jacc.2022.02.025. PMID: 35450580; PMCID: PMC9388228.
12. Chen C, Wen T, Liao W. Nocturnal supports for patients with central sleep apnea and heart failure: a systemic review and network meta analysis of randomized controlled trials. Ann Transl Med. 2019;7(14):337.
13. Farquhar JM, Stonerock GL, Blumenthal JA. Treatment of Anxiety in Patients With Coronary Heart Disease: A Systematic Review. Psychosomatics. 2018;59(4):318 32.
14. Afshari Saleh L, Asadpour H, Amini M, Hojjat S K, Talaei A, et al. Investigating of Mood and Sleep Disorders in Patients with Restless Legs Syndrome: A Cross Sectional Study.Jundishapur J Chronic Dis Care.2025;14(2):e158353.[https://doi.org/10.5812/jjcdc 158353](https://doi.org/10.5812/jjcdc-158353).
15. Kwekkeboom KL, Bratzke LC. A Systematic Review of Relaxation, Meditation, and Guided Imagery Strategies for Symptom Management in Heart Failure. J Cardiovasc Nurs. 2016;31(5):457 68.
16. Bhatt AS, DeVore AD, Hernandez AF, Mentz RJ. Can Vaccinations Improve Heart Failure Outcomes?: Contemporary Data and Future Directions. JACC Heart Fail. 2017;5(3):194 203.
17. Fountoulaki K, Tsiodras S, Polyzogopoulou E, Olympios C, Parissis J. Beneficial Effects of Vaccination on Cardiovascular Events: Myocardial Infarction, Stroke, Heart Failure. Cardiology. 2018;141(2):98 106.
18. Modin D, Jørgensen ME, Gislason G, Jensen JS, Køber L, Claggett B, et al. Influenza Vaccine in Heart Failure. Circulation. 2019;139(5):575 86.
19. Rørth R, Wong C, Kragholm K, Fosbøl EL, Mogensen UM, Lamberts M, et al. Return to the Workforce After First Hospitalization for Heart Failure: A Danish Nationwide Cohort Study. Circulation. 2016;134(14):999 1009.
20. Ismail H, McFarlane JR, Nojoumian AH, Dieberg G, Smart NA. Clinical outcomes and cardiovascular responses to different exercise training intensities in patients with heart failure: a systematic review and meta analysis. JACC Heart Fail. 2013;1(6):514 22.
21. Nolte K, Herrmann Lingen C, Wachter R, Gelbrich G, Düngen HD, Duvinage A, et al. Effects of exercise training on different quality of life dimensions in heart failure with preserved ejection fraction: the Ex DHF P trial. Eur J Prev Cardiol. 2015;22(5):582 93.
22. Taylor RS, Sagar VA, Davies EJ, Briscoe S, Coats AJ, Dalal H, et al. Exercise based rehabilitation for heart failure. Cochrane Database Syst Rev. 2014;2014(4):Cd003331.
23. Sepehrvand N, Alemayehu W, Rowe BH, McAlister FA, van Diepen S, Stickland M, et al. High vs. low oxygen therapy in patients with acute heart failure: HiLo HF pilot trial. ESC Heart Fail. 2019;6(4):667 77.
24. Wilhelm EAB, Davis LL, Sharpe L, Waters S. Assess and address: Screening and management of depression in patients with chronic heart failure. J Am Assoc Nurse Pract. 2022 May 1;34(5):769 779. doi: 10.1097/JXX.0000000000000701. PMID: 35383649.
25. Sohani ZN, Samaan Z. Does depression impact cognitive impairment in patients with heart failure? Cardiol Res Pract. 2012;2012:524325.
26. Jaarsma T, Hill L, Bayes Genis A, La Rocca HB, Castiello T, Čelutkienė J, Marques Sule E, Plymen CM, Piper SE, Riegel B, Rutten FH, Ben Gal T, Bauersachs J, Coats AJS, Chioncel O, Lopatin Y, Lund LH, Lainscak M, Moura B, Mullens W, Piepoli MF, Rosano G, Seferovic P, Strömberg A. Self care of heart failure patients: practical management recommendations from the Heart Failure Association of the European Society of Cardiology. Eur J Heart Fail. 2021 Jan;23(1):157 174. doi: 10.1002/ejhf.2008. Epub 2020 Oct 20. PMID: 32945600; PMCID: PMC8048442.
27. Diop MS, Rudolph JL, Zimmerman KM, Richter MA, Skarf LM. Palliative Care Interventions for Patients with Heart Failure: A Systematic Review and Meta Analysis. J Palliat Med. 2017;20(1):84 92.
28. Placino S, Ansay MFM, Mangubat GFE, Mahilum MLP, Al Abcha A, Suleman N, Shah N, Suboc TMB, Volgman AS. End Of Life Care for Patients With End Stage Heart Failure, Comparisons of International Guidelines. Am J Hosp Palliat Care. 2024 Jan;41(1):87 98. doi: 10.1177/10499091231154575. Epub 2023 Jan 27. PMID: 36705612.
29. Sidebottom AC, Jorgenson A, Richards H, Kirven J, Sillah A. Inpatient palliative care for patients with acute heart failure: outcomes from a randomized trial. J Palliat Med. 2015;18(2):134 42.
30. Golla MSG, Shams P. Heart Failure With Preserved Ejection Fraction (HFpEF) [Updated 2024 Mar 19]. In: StatPearls [Internet]. Treasure Island (FL): StatPearls Publishing; 2025 Jan . Available from: https://www.ncbi.nlm.nih.gov/books/NBK599960/
31. Chetran A, Costache AD, Ciongradi CI, Duca ST, Mitu O, Sorodoc V, Cianga CM, Tuchilus C, Mitu I, Mitea RD, Badescu MC, Afrasanie I, Huzum B, Moisa SM, Prepeliuc CS, Roca M, Costache II. ECG and Biomarker Profile in Patients with Acute Heart Failure: A Pilot Study. Diagnostics (Basel). 2022 Dec 3;12(12):3037. doi: 10.3390/diagnostics12123037. PMID: 36553044; PMCID: PMC9776598.
32. Breathett K, Allen LA, Udelson J, Davis G, Bristow M. Changes in Left Ventricular Ejection Fraction Predict Survival and Hospitalization in Heart Failure With Reduced Ejection Fraction. Circ Heart Fail. 2016;9(10).
33. Magdy JS, McVeigh J, Indraratna P. Diuretics in the management of chronic heart failure: when and how. Aust Prescr. 2022 Dec;45(6):200 204. doi: 10.18773/austprescr.2022.069. Epub 2022 Nov 30. PMID: 36479331; PMCID: PMC9722345.
34. Alcocer LA, Bryce A, De Padua Brasil D, Lara J, Cortes JM, Quesada D, Rodriguez P. The Pivotal Role of Angiotensin Converting Enzyme Inhibitors and Angiotensin II Receptor Blockers in Hypertension Management and Cardiovascular and Renal Protection: A Critical Appraisal and Comparison of International Guidelines. Am J Cardiovasc Drugs. 2023 Nov;23(6):663 682. doi: 10.1007/s40256 023 00605 5. Epub 2023 Sep 5. PMID: 37668854; PMCID: PMC10625506.
35. Sabina M, Shah S, Grimm M, Daher JC, Campillo P, Boozo MB, Al Abdouh A, Abusnina W, D' Ascenzo F, Bizanti A. Beta Blockers in Patients with Myocardial Infarction and Preserved Left Ventricular Ejection: A Systematic Review and Meta Analysis of Randomized Controlled Trials. J Clin Med. 2024 Dec 30;14(1):150. doi: 10.3390/jcm14010150. PMID: 39797234; PMCID: PMC11721919.
36. Dar JA, Jacob JR. Beta Blockers in Contemporary Cardiology: Is It Better to Cast Them Out?. Korean Circ J. 2024 Apr;54(4):165 171. <https://doi.org/10.4070/kcj.2023.0209>
37. Chourdakis E, Koniari I, Velissaris D, Tsigkas G, Kounis NG, Osman N. Beta blocker treatment in heart failure patients with atrial fibrillation: challenges and perspectives. J Geriatr Cardiol. 2021 May 28;18(5):362 375. doi: 10.11909/j.issn.1671 5411.2021.05.008. PMID: 34149825; PMCID: PMC8185444.
38. Fukuta H, Goto T, Kamiya T. Effects of calcium channel blockers in patients with heart failure with preserved and mildly reduced ejection fraction: A systematic review and meta analysis. Int J Cardiol Heart Vasc. 2024 Sep 19;55:101515. doi: 10.1016/j.ijcha.2024.101515. PMID: 39346950; PMCID: PMC11437750.
39. Gazewood JD, Turner PL. Heart Failure with Preserved Ejection Fraction: Diagnosis and Management. Am Fam Physician. 2017;96(9):582 8.
40. Hiraiwa, H., Okumura, T. & Murohara, T. Drug Therapy for Acute and Chronic Heart Failure with Preserved Ejection Fraction with Hypertension: A State of the Art Review. *Am J Cardiovasc Drugs* **24**, 343–369 (2024). https://doi.org/10.1007/s40256 024 00641 9
41. Ettehad D, Emdin CA, Kiran A, Anderson SG, Callender T, Emberson J, et al. Blood pressure lowering for prevention of cardiovascular disease and death: a systematic review and meta analysis. Lancet (London, England). 2016;387(10022):957 67.
42. Wright JT, Jr., Williamson JD, Whelton PK, Snyder JK, Sink KM, Rocco MV, et al. A Randomized Trial of Intensive versus Standard Blood Pressure Control. The New England journal of medicine. 2015;373(22):2103 16.
43. Olde Engberink RH, Frenkel WJ, van den Bogaard B, Brewster LM, Vogt L, van den Born BJ. Effects of thiazide type and thiazide like diuretics on cardiovascular events and mortality: systematic review and meta analysis. Hypertension (Dallas, Tex : 1979). 2015;65(5):1033 40.
44. Zeitler EP, Eapen ZJ. Anticoagulation in Heart Failure: a Review. Journal of atrial fibrillation. 2015;8(1).
45. Tscharre M, Mutschlechner D, Huber K, Gremmel T. Antiplatelet therapy with clopidogrel versus aspirin in atherosclerotic cardiovascular disease: a systematic review and meta analysis. Atherosclerosis. 2025 Oct;409:120478. doi: 10.1016/j.atherosclerosis.2025.120478. Epub 2025 Aug 15. PMID: 40845727.
46. Radmila Lyubarova, Joshua Schulman Marcus and William E. Boden. Contemporary Management of Patients with Stable Ischemic Heart Disease. CVIA. 2019. Vol. 3(3):269 278. DOI: 10.15212/CVIA.2017.0071
47. Savarese G, Giugliano RP, Rosano GM, McMurray J, Magnani G, Filippatos G, et al. Efficacy and Safety of Novel Oral Anticoagulants in Patients With Atrial Fibrillation and Heart Failure: A Meta Analysis. JACC Heart failure. 2016;4(11):870 80.
48. David MNV, Shetty M. Digoxin. [Updated 2024 Nov 25]. In: StatPearls [Internet]. Treasure Island (FL): StatPearls Publishing; 2025 Jan . Available from: https://www.ncbi.nlm.nih.gov/books/NBK556025/دوتاس دیگوکسین و نیماتد
49. Santangeli P, Muser D, Maeda S, Filtz A, Zado ES, Frankel DS, et al. Comparative effectiveness of antiarrhythmic drugs and catheter ablation for the prevention of recurrent ventricular tachycardia in patients with implantable cardioverter defibrillators: A systematic review and meta analysis of randomized controlled trials. Heart rhythm. 2016;13(7):1552 9.
50. Luigi Pannone, Giuseppe D’Angelo, Simone Gulletta, Giulio Falasconi, Luigia Brugliera, Antonio Frontera, Lorenzo Cianfanelli, Luca Baldetti, Paolo Ossola, Francesco Melillo, Gabriele De Blasi, Lorenzo Malatino, Giovanni Landoni, Alberto Margonato, Paolo Della Bella, Daniele Zacchetti, Pasquale Vergara. Amiodarone in ventricular arrhythmias: still a valuable resource?. Rev. Cardiovasc. Med. 2021, 22(4), 1383–1392. https://doi.org/10.31083/j.rcm2204143
51. Wang GM, Li LJ, Tang WL, Wright JM. Renin inhibitors versus angiotensin converting enzyme (ACE) inhibitors for primary hypertension. Cochrane Database Syst Rev. 2020;10(10):Cd012569.
52. Cohn JN, Johnson G, Ziesche S, Cobb F, Francis G, Tristani F, et al. A comparison of enalapril with hydralazine isosorbide dinitrate in the treatment of chronic congestive heart failure. N Engl J Med. 1991;325(5):303 10.
53. Jun HR, Kim H, Lee SH, Cho JH, Lee H, Yim HW, Yoon KH, Kim HS. Onset of Hyperkalemia following the Administration of Angiotensin Converting Enzyme Inhibitor or Angiotensin II Receptor Blocker. Cardiovasc Ther. 2021 Jan 22;2021:5935149. doi: 10.1155/2021/5935149. PMID: 33747123; PMCID: PMC7943268.
54. Pitak Arnnop P, Subbalekha K, Muangchan C, Auychai P, Sirintawat N, Meningaud JP, Neff A. Angiotensin Converting Enzyme Inhibitor Induced Non allergic Perioral Angioedema: A Case Based Scoping Review. Korean J Fam Med. 2023 Jan;44(1):2 10. doi: 10.4082/kjfm.21.0095. Epub 2021 Nov 23. PMID: 34808742; PMCID: PMC9887447.
55. Patel P, Launico MV. Angiotensin II Receptor Blockers (ARB) [Updated 2025 May 5]. In: StatPearls [Internet]. Treasure Island (FL): StatPearls Publishing; 2025 Jan . Available from: https://www.ncbi.nlm.nih.gov/books/NBK537027/
56. Khazanie P, Liang L, Curtis LH, Butler J, Eapen ZJ, Heidenreich PA, et al. Clinical Effectiveness of Hydralazine Isosorbide Dinitrate Therapy in Patients With Heart Failure and Reduced Ejection Fraction: Findings From the Get With The Guidelines Heart Failure Registry. Circ Heart Fail. 2016;9(2):e002444.
57. Sobhy M, Eletriby A, Ragy H, Kandil H, Saleh MA, Farag N, Guindy R, Bendary A, Nayel AME, Shawky A, Khairy A, Mortada A, Zarif B, Badran H, Khorshid H, Mahmoud K, Said K, Leon K, Abdelsabour M, Tawfik M, Abdelmegid MAF, Koriem M, Loutfi M, Wadie M, Elnoamany M, Sadaka M, Seleem M, Zahran M, Amin OA, Elkaffas S, Ayad S, Kilany WE, Ammar W, Elawady W, Elhammady W, Abdelhady Y. ACE Inhibitors and Angiotensin Receptor Blockers for the Primary and Secondary Prevention of Cardiovascular Outcomes: Recommendations from the 2024 Egyptian Cardiology Expert Consensus in Collaboration with the CVREP Foundation. Cardiol Ther. 2024 Dec;13(4):707 736. doi: 10.1007/s40119 024 00381 6. Epub 2024 Oct 25. PMID: 39455534; PMCID: PMC11607301.
58. Aronow WS, Ahn C, Kronzon I. Effect of beta blockers alone, of angiotensin converting enzyme inhibitors alone, and of beta blockers plus angiotensin converting enzyme inhibitors on new coronary events and on congestive heart failure in older persons with healed myocardial infarcts and asymptomatic left ventricular systolic dysfunction. Am J Cardiol. 2001;88(11):1298 300.
59. Strauss MH, Hall AS, Narkiewicz K. The Combination of Beta Blockers and ACE Inhibitors Across the Spectrum of Cardiovascular Diseases. Cardiovasc Drugs Ther. 2023 Aug;37(4):757 770. doi: 10.1007/s10557 021 07248 1. Epub 2021 Sep 17. PMID: 34533690; PMCID: PMC10397146.
60. Beer BN, Benson L, Basile C, Schrage B, Becher PM, Blankenberg S, Kirchhof P, Szabó Söderberg B, Metra M, Lindberg A, Imbalzano E, Rosano GMC, Karlström P, Mol PGM, Scorza R, Lund LH, Lindberg F, Savarese G. Beta blockers in patients with heart failure with reduced ejection fraction and concomitant chronic obstructive pulmonary disease: Cardiovascular and respiratory outcomes. Eur J Heart Fail. 2025 Sep 11. doi: 10.1002/ejhf.70046. Epub ahead of print. PMID: 40932340.
61. Rishi Sethi, Pravesh Vishwakarma, Akshyaya Pradhan, Evidence for Aldosterone Antagonism in Heart Failure, Cardiac Failure Review 2024;10:e15.
62. Wang TY, Vora AN, Peng SA, Fonarow GC, Das S, de Lemos JA, Peterson ED. Effectiveness and Safety of Aldosterone Antagonist Therapy Use Among Older Patients With Reduced Ejection Fraction After Acute Myocardial Infarction. J Am Heart Assoc. 2016 Jan 21;5(1):e002612. doi: 10.1161/JAHA.115.002612. PMID: 26796254; PMCID: PMC4859378.
63. Zannad F, McMurray JJ, Krum H, van Veldhuisen DJ, Swedberg K, Shi H, et al. Eplerenone in patients with systolic heart failure and mild symptoms. N Engl J Med. 2011;364(1):11 21.
64. Cohn JN, Archibald DG, Ziesche S, Franciosa JA, Harston WE, Tristani FE, et al. Effect of vasodilator therapy on mortality in chronic congestive heart failure. Results of a Veterans Administration Cooperative Study. N Engl J Med. 1986;314(24):1547 52.
65. Qavi AH, Kamal R, Schrier RW. Clinical Use of Diuretics in Heart Failure, Cirrhosis, and Nephrotic Syndrome. Int J Nephrol. 2015;2015:975934.
66. de Denus S, Rouleau JL, Mann DL, Huggins GS, Cappola TP, Shah SH, et al. A pharmacogenetic investigation of intravenous furosemide in decompensated heart failure: a meta analysis of three clinical trials. Pharmacogenomics J. 2017;17(2):192 200.
67. Faris R, Flather M, Purcell H, Henein M, Poole Wilson P, Coats A. Current evidence supporting the role of diuretics in heart failure: a meta analysis of randomised controlled trials. International journal of cardiology. 2002;82(2):149 58.
68. Slatton ML, Irani WN, Hall SA, Marcoux LG, Page RL, Grayburn PA, et al. Does digoxin provide additional hemodynamic and autonomic benefit at higher doses in patients with mild to moderate heart failure and normal sinus rhythm? Journal of the American College of Cardiology. 1997;29(6):1206 13.
69. Ziff OJ, Lane DA, Samra M, Griffith M, Kirchhof P, Lip GY, et al. Safety and efficacy of digoxin: systematic review and meta analysis of observational and controlled trial data. BMJ (Clinical research ed). 2015;351:h4451.
70. Cao C, Martinelli A, Spoelhof B, Llinas RH, Marsh EB. In Potential Stroke Patients on Warfarin, the International Normalized Ratio Predicts Ischemia. Cerebrovascular diseases extra. 2017;7(2):111 9.
71. Cokkinos DV, Haralabopoulos GC, Kostis JB, Toutouzas PK. Efficacy of antithrombotic therapy in chronic heart failure: the HELAS study. European journal of heart failure. 2006;8(4):428 32.
72. Nemerovski CW, Salinitri FD, Morbitzer KA, Moser LR. Aspirin for primary prevention of cardiovascular disease events. Pharmacotherapy. 2012;32(11):1020 35.
73. Farré J, Romero J, Rubio JM, Ayala R, Castro Dorticós J. Amiodarone and "primary" prevention of sudden death: critical review of a decade of clinical trials. The American journal of cardiology. 1999;83(5b):55d 63d.
74. Javot L, Pape E, Yéléhé Okouma M, Barotte E, Divoux E, Gillet P, et al. Intravenous single administration of amiodarone and breastfeeding. Fundamental & clinical pharmacology. 2019;33(3):367 72.
75. Skeoch S, Weatherley N, Swift AJ, Oldroyd A, Johns C, Hayton C, et al. Drug Induced Interstitial Lung Disease: A Systematic Review. Journal of clinical medicine. 2018;7(10).
76. Makani H, Bangalore S, Desouza KA, Shah A, Messerli FH. Efficacy and safety of dual blockade of the renin angiotensin system: meta analysis of randomised trials. BMJ (Clinical research ed). 2013;346:f360.
77. Dickstein K, Kjekshus J. Effects of losartan and captopril on mortality and morbidity in high risk patients after acute myocardial infarction: the OPTIMAAL randomised trial. Optimal Trial in Myocardial Infarction with Angiotensin II Antagonist Losartan. Lancet (London, England). 2002;360(9335):752 60.
78. Yusuf S, Sleight P, Pogue J, Bosch J, Davies R, Dagenais G. Effects of an angiotensin converting enzyme inhibitor, ramipril, on cardiovascular events in high risk patients. The New England journal of medicine. 2000;342(3):145 53.
79. Nyolczas N, Dékány M, Muk B, Szabó B. Combination of Hydralazine and Isosorbide Dinitrate in the Treatment of Patients with Heart Failure with Reduced Ejection Fraction. Advances in experimental medicine and biology. 2018;1067:31 45.
80. McMurray JJ, Ostergren J, Swedberg K, Granger CB, Held P, Michelson EL, et al. Effects of candesartan in patients with chronic heart failure and reduced left ventricular systolic function taking angiotensin converting enzyme inhibitors: the CHARM Added trial. Lancet (London, England). 2003;362(9386):767 71.
81. Cohn JN, Ziesche S, Smith R, Anand I, Dunkman WB, Loeb H, et al. Effect of the calcium antagonist felodipine as supplementary vasodilator therapy in patients with chronic heart failure treated with enalapril: V HeFT III. Vasodilator Heart Failure Trial (V HeFT) Study Group. Circulation. 1997;96(3):856 63.
82. Meems LM, van der Harst P, van Gilst WH, de Boer RA. Vitamin D biology in heart failure: molecular mechanisms and systematic review. Current drug targets. 2011;12(1):29 41.
83. Conti CR, Pepine CJ, Sweeney M. Efficacy and safety of sildenafil citrate in the treatment of erectile dysfunction in patients with ischemic heart disease. The American journal of cardiology. 1999;83(5a):29c 34c.
84. Redfield MM, Chen HH, Borlaug BA, Semigran MJ, Lee KL, Lewis G, et al. Effect of phosphodiesterase 5 inhibition on exercise capacity and clinical status in heart failure with preserved ejection fraction: a randomized clinical trial. Jama. 2013;309(12):1268 77.
85. Scott PA, Kingsley GH, Scott DL. Non steroidal anti inflammatory drugs and cardiac failure: meta analyses of observational studies and randomised controlled trials. European journal of heart failure. 2008;10(11):1102 7.

**Supplementary File 4**

**Table 3.** Common Medications used in the Management of Heart Failure Patients

| **Drug Class** | **Medications** |
| --- | --- |
| ACE Inhibitors | Captopril, Lisinopril, Enalapril |
| Angiotensin II Receptor Blockers (ARBs) | Losartan, Valsartan |
| Beta blockers | Carvedilol, Metoprolol succinate, Bisoprolol |
| Diuretics | Furosemide, Triamterene, Hydrochlorothiazide |
| Aldosterone Antagonists | Spironolactone (Aldactone), Eplerenone |

**Supplementary File 5**

**Table 4.** Adverse Drug Reactions in Patients with Chronic Heart Failure

| **Drug / Drug Class** | **Common Adverse Effects** | **Serious or Life Threatening Adverse Effects** |
| --- | --- | --- |
| Diuretics | Orthostatic hypotension, urinary urgency | Electrolyte imbalance (hypokalemia, hypomagnesemia, hyponatremia), arrhythmia |
| ACE inhibitors | Cough, orthostatic hypotension | Worsening renal function, angioedema |
| Beta blockers | Bradycardia, fatigue, cold extremities | Asthma attacks, heart block, worsening heart failure |
| Aldosterone antagonists (Spironolactone, Eplerenone) | Gynecomastia, fatigue, skin rash | Hyperkalemia, hyponatremia |
| Digoxin^[1]^ | Nausea | Life threatening arrhythmias |
| Angiotensin II receptor blockers (ARBs) | Orthostatic hypotension | Worsening renal function |
| Amiodarone | Photosensitivity, nausea, thyroid dysfunction, sleep disturbances, corneal microdeposits | Hepatic or pulmonary fibrosis, thyroid storm, pro arrhythmia |
| Inotropes | Nausea, abdominal pain | Arrhythmia, cardiotoxicity |

1. Digoxin Use in Heart Failure Management
   Digoxin may be considered in patients with systolic heart failure or heart failure accompanied by

   supraventricular arrhythmias such as atrial fibrillation.
   If a patient’s symptoms are well controlled with digoxin therapy and no adverse effects are observed, discontinuation of the drug is not recommended, even in cases of diastolic heart failure.

   Optimization of Therapy:
   The therapeutic effect of digoxin can be enhanced by:

   - Adding a diuretic, provided that potassium balance is maintained;
   - Combining with an ACE inhibitor or an angiotensin receptor blocker (ARB);
   - Or co-administration with a vasodilator, when appropriate.

   Pharmacokinetic Considerations:
   Due to its long half life, digoxin should be administered once daily.
   To prevent accumulation and potential toxicity, it is recommended that patients take the medication five days per week, with two non-consecutive drug free days (e.g., Mondays and Fridays).
   Dose adjustment is required in patients with renal impairment.

   Drug interactions with digoxin are primarily pharmacodynamic rather than metabolic, and require careful clinical monitoring.
